# Hippo Pathway Counter-Regulates Innate Immunity in Hepatitis B Virus Infection

**DOI:** 10.3389/fimmu.2021.684424

**Published:** 2021-05-25

**Authors:** Xufeng Luo, Rui Zhang, Mengji Lu, Shi Liu, Hideo A. Baba, Guido Gerken, Heiner Wedemeyer, Ruth Broering

**Affiliations:** ^1^ Institute for Lymphoma Research, Henan Cancer Hospital, The Affiliated Cancer Hospital of Zhengzhou University, Zhengzhou University, Zhengzhou, China; ^2^ Department of Gastroenterology and Hepatology, University Hospital Essen, University of Duisburg-Essen, Essen, Germany; ^3^ Department of Biliary-Pancreatic Surgery, Sun Yat-sen Memorial Hospital, Sun Yat-sen University, Guangzhou, China; ^4^ Institute for Virology, University Hospital Essen, University of Duisburg-Essen, Essen, Germany; ^5^ State Key Laboratory of Virology, Modern Virology Research Center, College of Life sciences, Wuhan University, Wuhan, China; ^6^ Institute for Pathology, University Hospital Essen, University of Duisburg-Essen, Essen, Germany; ^7^ Department of Gastroenterology, Hepatology and Endocrinology, Hannover Medical School, Hannover, Germany

**Keywords:** HBV, TLR - toll-like receptor, innate immunity, Hippo, IkBalpha

## Abstract

Whether hepatitis B virus (HBV) activates or represses innate immunity continues to be debated. Toll-like receptor (TLR) 2 has been identified to recognize HBV particles in human hepatocytes. The Hippo pathway, known for growth control, is suggested to play a vital role in immune regulation. Here, molecular interactions between HBV-triggered TLR signaling and the Hippo pathway were comprehensively investigated. Reanalysis of GSE69590 data, in which human hepatocytes have been treated with cell culture-derived HBV particles, identified changes in Hippo and NF-κB signaling. Immunocytochemical staining and western blotting revealed time-dependent nuclear translocation of YAP and NF-κB in HBV-exposed primary human and murine hepatocytes (PMH). Analysis of PMH isolated from MyD88- or IRAK4-deficient mice and the inhibition of TLR2 and MST1/2 *in vitro* confirmed the relation between TLR2 and Hippo signaling in HBV-induced immunity. Loss and gain of function experiments implied that Hippo-downstream effector YAP directly regulated IκBα expression. Functional investigations confirmed the regulation of *Nfkbia* promoter activity by the YAP/TEAD4 transcription factor complex. Administration of TLR ligands to mice highlighted the relevance of the TLR2-MyD88-IRAK4-Hippo axis in hepatic immunity. Interestingly, reanalysis of gene expression pattern in liver biopsies of patients chronically infected with HBV (GSE83148, GSE65359) indicated an activation of TLR2 and however, an MST1-dominated Hippo control in the immune clearance phase of patients with chronic HBV infection. We demonstrated that MyD88-dependent TLR signaling activates NF-κB and Hippo signaling, with YAP prompting the IκBα-mediated negative feedback, alongside NF-κB. Imbalance between immune induction and Hippo activation may have implications for the safety of novel HBV cure strategies interfering with pathogen recognition receptors.

## Introduction

Hepatitis B virus (HBV) infection seriously threatens human health. Globally, approximately 2 billion people have been infected with HBV, with more than 290 million chronic HBV infections (1). Even though a variety of mechanisms promoting prevention and control of HBV infection have been characterized during the past decades, the detailed role of innate immunity in acute and chronic HBV infection is controversially discussed. Most viral infections can be surveilled and detected by pattern recognition receptors (2), which induce antiviral innate responses, including interferons and inflammatory cytokines. However, no interferon-related innate response has been observed in diverse HBV infection models (3–5), which implies that HBV acts as a “stealth virus” that eludes interferon-based responses. Interestingly, Yoneda et al. showed NF-κB activation in HBV-treated human hepatocytes (6). Accordingly, our previous work suggested that HBV is recognized by TLRs in primary murine hepatocytes (PMHs) (7). Most recently, TLR2 has been identified to sense HBV in primary human hepatocytes (PHHs) (8), leading to inflammatory gene expression through NF-κB-related signaling pathways. Fundamental immunological mechanisms, especially those of immune escape and immune control in chronic HBV infection, remain unclear.

The NF-κB family is a key response element involved in innate and adaptive immune responses. The mammalian NF-κB family has many members, which includes RELA (p65), NF-κB1 (p50; p105), NF-κB2 (p52; p100), c-REL and RELB (9). NF-κB proteins are present in the cytoplasm in association with inhibitory proteins that are known as inhibitors of NF-κB (IκBs), of which the most common are IκBα, IκBβ and IκBϵ (10). It has been well documented that IκBα regulates transient NF-κB activation and that IκBβ maintains persistent NF-κB activation (11). IκBα is rapidly proteolyzed in response to stimuli and quickly resynthesized, owing to the presence of an NF-κB response element in its promoter (12). The newly synthesized IκBα has an intrinsic nuclear-localization sequences. Therefore IκBα enters the nucleus, displaces NF-κB from its DNA binding sites and transports NF-κB to the cytoplasm (9). Furthermore, only IκBα, but not IκBβ, contains a functional nuclear-export signal at its amino terminus, which is essential for shuttling the NF-κB–IκBα complex (9). The liver represents a complex inflammatory microenvironment. The hepatic immune system on the one hand tolerates harmless molecules while on the other hand induces adequate responses to infectious agents or tissue damage. Resolving inflammation is mandatory for maintaining liver homeostasis. Inflammatory imbalance can lead to the development of fibrosis, cirrhosis and hepatocellular carcinoma (13).

The Hippo pathway, originally discovered in *Drosophila*, controls cell proliferation and organ size (14, 15). Hippo signaling is highly conserved in mammals, MST1/2, SAV1, LATS1/2 and MOB1 represent the core kinase cascade (16, 17) and control YAP nuclear translocation and YAP/TEAD transcription factor complex formation (18, 19). Interestingly, Liu et al. showed that Hpo (MST orthologue) is activated rapidly through the Toll receptor in *Drosophila* after gram-positive bacterial infection (20). Moreover, TLRs recognize bacterial infection and mediate the activation of MST1/2 to control the production of reactive oxygen species for bacterial clearance in mice (21). Hippo signaling not only functions in antibacterial innate responses but also plays an important role in antiviral defense. As part of a cytosolic effector complex YAP directly interferes with TBK1 and abolishes virus-induced TBK1 activation (22). Thus, Hippo signaling seems to play a vital role in immune regulation.

**Figure 1:**
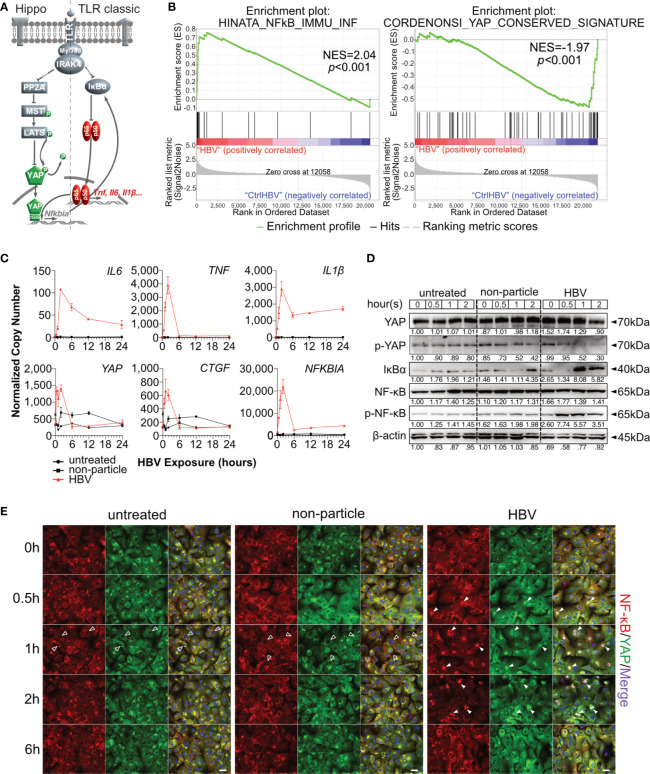
Hippo signaling is affected during HBV-induced innate immunity in primary human hepatocytes. Hypothesis of TLR-associated Hippo pathway activation **(A)**. Gene set enrichment analysis of GSE69590 comparing HBV-infected primary human hepatocytes (40h) and mock control gene sets **(B)**. Primary human hepatocytes (PHHs) from four different donors were treated with cell culture-derived HBV (MOI of 1,000) for different lengths of time. Gene expression of *IL6*, *TNF*, *IL1β*, *YAP*, *CTGF* and *NFKBIA* was determined by quantitative PCR **(C)**, (normalized to *ACTB*, mean ± SEM, n=4). Western blotting was performed to determine the expression of PP2A, phosphorylated PP2A, MyD88, MST1/2, phosphorylated MST1/2, YAP, phosphorylated YAP, IκBα, NF-κB and phosphorylated NF-κB after the exposure in PHHs to HBV. Gel images were obtained with ADVANCED Fluorescence and ECL Imager, representative of n=4 **(D)**. Representative ICC staining images were obtained with Zeiss AxioObserver.Z1 and Apotome (40x objective) to visualize the intracellular localization **(E)** of YAP (green) and NF-κB (red), raw images were subsequently proceeded with Image J software, n=3 independent experiments. Arrow heads highlight nuclear exclusion (empty arrows) or nuclear translocation (full arrows) of transcription factors. Scale bar, 20μm; kDa, kilodalton.

Several novel HBV cure strategies are currently explored including the activation of innate immune pathways (23). A more detailed understanding on innate immune induction and intracellular regulation is important for the comprehension of treatment safety and efficacy of these immune modulators (23–25). Here, we report for the first time that HBV particles that are recognized by TLRs on murine (7) and human hepatocytes (8) concordantly activated NF-κB and Hippo signaling. This activation led to the rapid induction of innate immune response and control of the innate host defense, respectively. Interestingly, the classic activation of NF-κB was accompanied by YAP nuclear translocation, identified here as an additional transcriptional inducer of IκBα expression, balancing the NF-κB signaling intensity (**Figure 1A**). In patients, chronically suffering from HBV infection, hepatic gene signatures confirmed the relation between TLR and Hippo pathways. Our data revealed that the Hippo pathway regulates HBV-induced innate immunity, linking the processes of inflammation and growth control.

## Materials and Methods

### HBV Particles and Non-Particle Control Preparation

HBV particles were prepared from hepatoma cells, stably transfected with an HBV-coding plasmid (HepG2.117 cell line), as previously described (8). Briefly, HBV particles (genotype D, serotype ayw) were prepared from cell culture supernatants by overnight precipitation with 6% polyethylene glycol 8000 (PEG8000; Sigma, Darmstadt, Germany) at 4°C, concentrated by centrifugation (12,000g for 60 minutes at 4°C) and stored at −80°C. HBV stocks contained 10^9^ genome equivalents/ml. The non-particle control was produced by precipitating supernatants of HepG2 cells under the same conditions.

### Primary Human Hepatocyte Isolation and Stimulation

Primary human hepatocytes were prepared from non-tumorous tissue obtained from freshly resected livers of four different donors, as previously described (26). PHHs were seeded into collagen I-coated culture plates or coverslips and stimulated with cell culture-derived HBV (MOI of 1,000) or a non-particle control, one day after preparation (8). All patients provided written documentation of their informed consent. The study conformed to the ethical guidelines of the 1975 Declaration of Helsinki and was approved by the Institutional Review Board (Ethics Committee) of the medical faculty at the University Duisburg-Essen. Human biological samples and related data were provided by the *Westdeutsche Biobank Essen* (WBE, University Hospital Essen, University of Duisburg-Essen, Essen, Germany; approval 18-WBE-048).

### Mouse Experiments

Mouse experiments were performed according to the Institutional Animal Care and Use Committee guidelines of the Animal Core Facility of University Hospital Essen. Mice were bred at University Hospital Essen and given humane care according to the criteria outlined in the Guide for the Care and Use of Laboratory Animals prepared by the Society for Laboratory Animal Science. Ultrapure LPS (2.5mg/kg bodyweight) or Pam3CSK4 (1mg/kg bodyweight) (Invivogen, San Diego, USA) were diluted in PBS and injected i.p. or i.v., respectively. In total, 12 mice were used for injection with Pam3CSK4 or LPS, with a group size of n=3 mice for each time point (0h, 1h, 2h and 6h).

### Primary Murine Hepatocyte Isolation and Stimulation

PMHs were prepared from wildtype C57BL/6 (6-9 months-old, male) mice, *Irak4^-/-^* mice and *Myd88^-/-^/Trif^-/-^* mice by *in-situ* collagenase type IV (Worthington, Lakewood, USA) perfusion, as described previously (27); due to TLR2/4 context, in the following the *Myd88^-/-^/Trif^-/-^* mice are termed *Myd88^-/-^* mice. Knockout mice were kindly provided by Prof. Carsten Kirschning. PMHs were seeded into collagen I-coated culture plates or coverslips. One day after their preparation, PMHs were stimulated with cell culture-derived HBV (MOI of 1,000) or a non-particle control. HBV particle preparation and stimulation were performed as previously described (7).

### Cell Culture and Transfection

The Hepa1-6, NCTC clone 1469 and HEK293T cell lines were maintained in DMEM (Gibco, USA) containing L-Glutamine and supplemented with 10% FBS (Millipore, Darmstadt, Germany) and 1% penicillin/streptomycin (Millipore) at 37°C in a 5% CO_2_ incubator. FuGENE HD (Promega, Madison, USA) and HiPerFect Transfection Reagent (Qiagen, Hilden, Germany) were used for plasmid and siRNA transfection of cell lines, respectively. In PMH Lipofectamine LTX and Lipofectamine RNAiMAX (Invitrogen, Vilnius, Lithuania) were used.

### siRNA, shRNA and Reagents

All siRNAs were synthesized by GenePharma (Shanghai, China). 2’-O-methylation prevents off-target immune induction. The shRNA target sequences were the same as those of the siRNAs (**Supplementary Table 1**). The shRNA sequences were cloned into psiRNA-h7SK-G1-GFPzeo plasmid (InvivoGen, San Diego, USA) and stably transfected into the Hepa1-6 cell line. GFP-positive clones were selected in the presence of zeocin (1000µg/ml) (InvivoGen).

### Chromatin Immunoprecipitation

The ChIP assay was performed with an EZ ChIP kit (Merck, Darmstadt, Germany) following the user guide as described previously (28). All ChIP primers were listed in **Supplementary Table 2**.

### Electrophoretic Mobility Shift Assay

EMSA was performed as described previously (29). To facilitate the assay, the radioisotope was replaced with GelRed (Biotium, Fremont, USA).

### Immunocytochemistry

Tissue specimens were fixed and paraffin embedded. Slides were deparaffinized, unmasked and blocked with 5% BSA in PBS. Cultured cells were washed 3 times with PBS and fixed for 10min at room temperature with 4% paraformaldehyde. Cells were additionally washed with PBS, after which immunocytochemical staining was conducted. Fixed cells or tissue slides were incubated for 10min on ice with 0.2% Triton X-100 (Sigma-Aldrich, Steinheim, Germany) and 0.2% BSA (Carl Roth, Karlsruhe, Germany) in PBS. Following permeabilization, the cells were blocked by incubation for 1h at room temperature with 0.02% Triton X-100 and 5% BSA in PBS. Primary antibodies were applied overnight at 4°C. Cells were washed with PBS three times and incubated with secondary antibodies at room temperature for 1h without light exposure. Finally, the cells were washed three times with PBS and covered with Fluoroshield™ mounting medium, including DAPI (Sigma-Aldrich).

### Sample Preparation

To prepare total mRNAs, QIAzol Lysis Reagent (Qiagen) was used to lyse and homogenize samples based on the manufacturer’s instructions. To prepare total protein lysates, RIPA buffer (150 mM NaCl, 1% Nonidet P-40, 0.5% sodium deoxycholate, 0.1% SDS, 25 mM Tris) supplemented with protease and phosphatase inhibitor cocktail (Thermo Scientific, USA) was used. Lysates were centrifuged for 15min at 14,400×g and 4°C. The protein concentration was determined with BCA Protein Assay Kit (Pierce, Rockford, USA). Samples were prepared in 5× loading buffer and incubated at 95°C for 5min.

### Quantitative Real-Time PCR

To analyze mRNA levels, 2μg of total RNA was reverse transcribed with SMART^®^ MMLV Reverse Transcriptase (TaKaRa, Kusatsu, Japan). The cDNA was diluted and mixed with FS Universal SYBR Green Master (Roche, Mannheim, Germany) and gene-specific primers (**Supplementary Table 3**). PCR was performed with a CFX96 Touch™ Real-Time PCR Detection System (Bio-Rad, Hercules, USA). The PCR results were analyzed with CFX Manager™ Software.

### Western Blotting

Twenty micrograms of total protein were loaded on 10% resolving and 5% stacking gel and resolved with a Mini-PROTEAN^®^ Tetra Cell (Bio-Rad) and transferred onto PVDF membranes (Trans-Blot^®^ Turbo™, BioRad). Primary and secondary antibodies used are listed in **Supplementary Table 4**. Amersham ECL Prime Western Blotting Detection Reagent (GE Healthcare, Chicago, USA) and an ADVANCED Fluorescence and ECL Imager (INTAS, Göttingen, Germany) were used to visualize light signals.

### Dual-Luciferase Reporter Assay

Assays were performed with pGL3-basic reporter plasmid (Promega, Madison, USA) and pRL-TK control plasmid (Promega). *Nfkbia* promoter region (-977 ~ +34, NM_010907), a truncated promoter region and a mutant TEAD4-binding site fragment were cloned into the multiple cloning site. To visualize *Nfkbia*-driven activity, red fluorescent protein (RFP) was cloned into the multiple cloning site (pRFP-Nfkbia). Cells of 80% confluence were transfected with reporter plasmids and/or overexpression plasmids as indicated. The Dual-Luciferase^®^ Reporter Assay System (Promega) was used to detect Firefly (control signal) and Renilla luciferase activities with a FLUOstar Omega microplate reader (BMG LABTECH, Ortenberg, Germany) 48h after transfection. The shRNA-GFP-positive Hepa1-6 clones were transfected with pRFP-Nfkbia for an additional 48h in chamber slides. The slides were fixed with 4% PFA and RFP signals were visualized with a BX53 upright Microscope (Olympus) or an AxioObserver. Z1 inverted microscope (ZEISS). RFP-positive cells were counted with a CytoFLEX S (Beckman, Brea, USA).

### Protein Expression and Purification

Murine *Tead4* sequence (NM_001080979) was cloned into the His-GST-TEV-pET(2G-T) vector, producing hexahistidine (His_6_)-GST-tagged proteins. Recombinant TEAD4 was expressed in *E. coli* strain BL21 (DE3). The protein was bound to high-capacity Glutathione Sepharose 4B GST-tagged protein purification resin (GE Healthcare) according to the manufacturer’s instructions. Recombinant TEAD4 was eluted (50mM Tris, 10mM reduced glutathione, pH 8.0) from the resin and stored at 4°C.

### Gene Expression Omnibus (GEO) Microarray Datasets

GSE69590 (6), GSE83148 (30) and GSE65359 (31) were reanalyzed by gsea-3.0 and R software to generate enriched signaling and fold-changed genes, respectively. Genset enrichment analysis (GSEA) was performed using the GEO matrix, containing the log2-values of all samples. Enrichment scores were calculated for gen sets predefined by the Molecular Signatures Database collections (i.e. C6 - oncogenic signature gene sets, Hallmark and KEGG). The Y-axis indicated ranked genes as signal-to-noise ratio (Signal2Noise), calculating the difference between the mean of treated vs untreated samples and additive product of standard deviations in treated and untreated samples. Selected gene sets were visualized by enrichment plots and heatmaps. In GSE69590, PHHs were infected with HBV particles (MOI=50) or remained untreated for 40h to characterize the role of DNA sensing pathways in the liver. In GSE83148, 122 HBV-infected liver tissues and 6 uninfected liver samples were utilized to describe the gene expression features in patients with chronic HBV infection. In GSE65359, the transcriptomes of 83 chronic hepatitis B patients (22 immune tolerant, 50 immune clearance, and 11 inactive carrier) were analyzed by performing microarray analysis of liver biopsies to describe the gene expression features in patients with different clinical phases.

### Data Acquisition, Statistical Analysis, and Software

ImageJ software was used to process high resolution images. RFP- and GFP-positive cells were quantified by flow cytometry. Reanalysis of gene expression omnibus data GSE69590 (6), GSE83148 (30) and GSE65359 (31), Gene Set Enrichment Analysis (gsea-3.0) and R project (Version 3.6.1) were utilized. Data analysis, linear models and differential expression was processed *via* limma package version 3.9. Student’s *t*-test was used to statistically indicate differences between two groups. Significance levels were defined as follows: *p<0.05, **p<0.01, and ***p<0.001, ****p<.0001 (Prism7). Representative data from a series of at least three independent experiments carried out in triplicate are presented as the mean ± standard error of the mean (SEM) unless otherwise indicated.

## Results

### HBV-Induced Innate Immune Responses Involve Hippo Signaling

Hippo signaling pathway has been found to play an important role in innate immunity. To evaluate the potential role of Hippo signaling in innate immunity during HBV infection, microarray data (GSE69590) (6) from PHH, infected with HBV for 40h, were reanalyzed. Significant enrichment of NF-κB-associated genes was observed in HBV-infected PHHs (p<0.001), while YAP-associated genes were enriched in the treatment control (p<0.001, **Figure 1B**). Heatmaps indicate the gene IDs and signal intensities among these annotations (**Supplementary Figure 1**). To address a possible link between innate and Hippo signaling, PHHs were exposed to HBV particles or non-particle control for different lengths of time. Gene expression levels of *IL6*, *TNF*, *IL1β* increased according to our previous work (8). Interestingly *YAP*, *CTGF* and *NFKBIA* gene expression also increased after HBV particle exposure at the very early stage (**Figure 1C**), compared to untreated and the non-particle control-treated PHH. Generally, YAP is excluded from the nucleus through its constitutive phosphorylation, leading to the decrease of its target genes. Dephosphorylation of YAP results in nuclear translocation and formation of the YAP/TEAD complex to regulate target gene expression (**Figure 1A**). Western blotting showed that phosphorylated YAP sharply declined after 1h and phosphorylated NF-κB already appeared 30min after treatment with HBV particles. The very early disappearance of IκBα and its immediate rebound indicated a rapid immune regulation (**Figure 1D**). Finally, ICC staining illustrated that HBV particles were recognized by PHHs and induced NF-κB and YAP nuclear translocation to induce and regulate the innate immune response, respectively (**Figure 1E**). These results indicate an immediate Hippo inactivation and Yap nuclear translocation after innate recognition of HBV particles by human hepatocytes. Our previous work (8) included appropriate functional controls. HBV-induced TLR2 signaling not only induce the expression of cytokine genes, but also leads to production and secretion of IL6, TNF and IL1β. Furthermore, ultraviolet irradiation of viral particles suppresses HBV infectivity but not the induction of cytokines in PHH, suggesting that the inoculum contains the immune-inducing agent. Purified HBV particles on the whole, which have been prepared from HBV DNA-positive and protein-rich fractions after heparin column separation, still have immune-inducing capacity in PHH (8), excluding the impact of contaminants in HBV particle-induced TLR2 activation.

**Figure 2:**
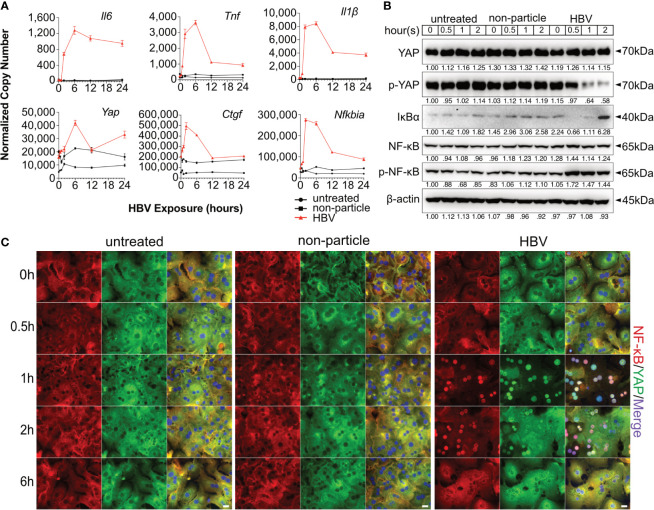
Hippo and NF-κB signaling are induced by HBV exposure in primary murine hepatocytes. Primary murine hepatocytes (PMHs, n=3) were treated with cell culture-derived HBV (MOI of 1,000) or non-particle control for different time points. Gene expression of *Il6, Tnf, Il1β, Yap, Ctgf* and *Nfkbia* was determined by quantitative PCR **(A)**, (normalized to *Actb*, mean ± SEM). Western blotting was performed to detect YAP, phosphorylated YAP, IκBα, NF-κB and phosphorylated NF-κB in HBV-exposed PMHs. Gel images were obtained with ADVANCED Fluorescence and ECL Imager, representative of n=3 independent experiments **(B)**. Representative ICC staining was performed and obtained with Zeiss AxioObserver.Z1 and Apotome (40x objective) to visualize YAP (green) and NF-κB (red) localization, raw images were subsequently proceeded with Image J software, representative of n=3 independent experiments **(C)**. Scale bar, 20μm; kDa, kilodalton.

PMHs can be homogeneously reproduced and were used to verify this observation. A switch to the mouse system enabled the analysis of knockout strains and *in vivo* experiments, as described later. Our previous work showed that HBV particles can induce innate immune responses in PMHs (7), although no infection process occurs. Furthermore, HBV dose- dependently (MOI 62.5-1,000) induced the expression of *IL1B*, *IL6* and *TNF*, whereas the mock control (MOI 500/1,000 equivalents) did not affect cytokine gene expression (8). Here, PMHs were treated with cell culture-derived HBV particles (multiplicity of infection [MOI] of 1,000) or non-particle control (equivalent volume) for different lengths of time (0.5 - 24h). A marked increase in *Il6*, *Tnf* and *Il1β* expression was observed 1h after HBV treatment and peaked at 6h (**Figure 2A**). YAP is a critical transcription factor downstream of the Hippo signaling pathway, its own transcription level was only slightly induced by the treatment with HBV particles (6h, p<0.001) compared to the non-particle control. While its target gene, *Ctgf*, was remarkably induced (2h, p<0.001). Expression of *Nfkbia*, the essential inhibitor of NF-κB signaling, was induced by HBV as well (**Figure 2B**). As shown for the human hepatocytes, HBV-exposed PMH showed decreased level of phosphorylated YAP compared to untreated or non-particle controls, indicating activation of the YAP/TEAD complex upon HBV exposure (**Figure 2B**). Simultaneously, HBV exposure increased the level of phosphorylated NF-κB and decreased the total IκBα level. Interestingly, IκBα expression was increased after YAP nuclear translocation, which raises the question whether *Nfkbia* expression might be regulated by YAP. Nuclear translocation of YAP and NF-κB in HBV-exposed PMHs was visualized by ICC staining (**Figure 2C**). YAP and NF-κB translocated into the nucleus 1-2h after HBV exposure. Consistent with the western blotting results, exposure of PMHs to HBV not only activated NF-κB signaling but also affected the Hippo pathway. These findings indicate, that PMH responded to HBV particle exposure in the same way as PHH do.

### TLR2-Mediated Activation of Hippo Signaling Is Essential to Control HBV-Induced Innate Responses

**Figure 3:**
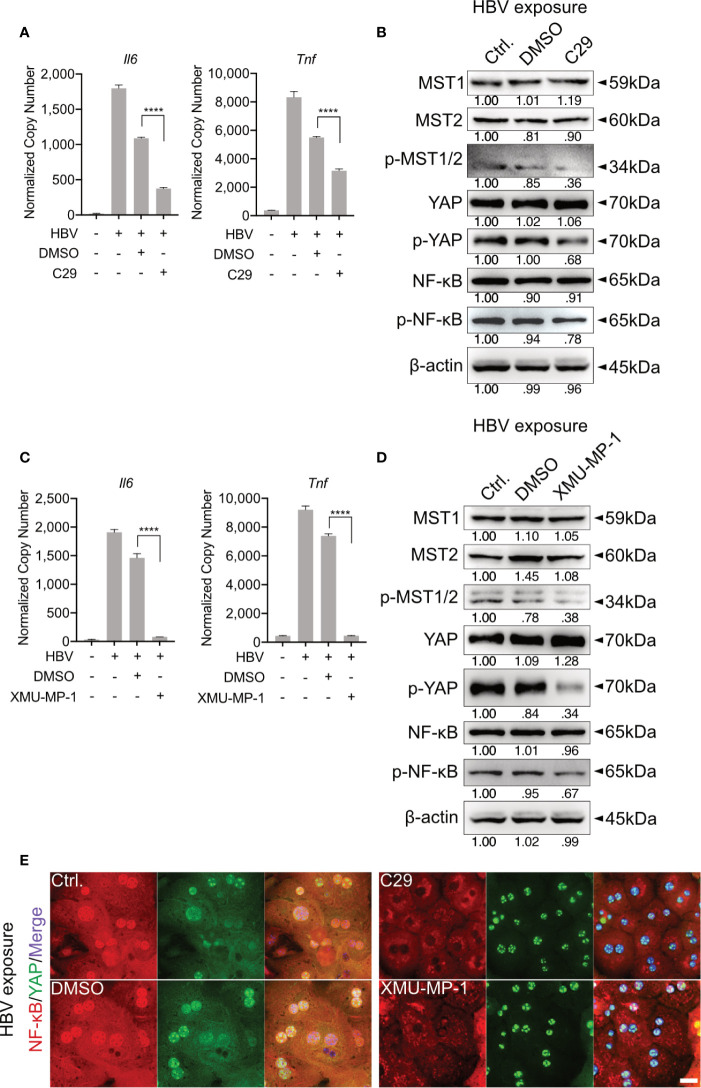
TLR2-mediated activation of Hippo signaling is essential for the control of rapid innate responses after HBV exposure. Primary murine hepatocytes (PMHs) were treated with TLR2 inhibitor C29 [50μM] or MST1/2 inhibitor XMU-MP-1 [1μM] for 2h prior to HBV exposure for an additional 2-6h. At 6h quantitative PCR was performed to detect *Il6* and *Tnf* expression in C29-pretreated **(A)** and XMU-MP-1-pretreated **(C)** PMHs (normalized to *Actb*, mean ± SEM, n=3 independent experiments). Western blotting was performed to detect MyD88, MST1/2, phosphorylated MST1/2, YAP, phosphorylated YAP, NF-κB and phosphorylated NF-κB in C29-pretreated **(B)** and XMU-MP-1-pretreated **(D)** PMHs 2h after HBV exposure, representatively. Gel images were obtained with ADVANCED Fluorescence and ECL Imager, n=3 independent experiments. ICC staining was performed and visualized using Zeiss AxioObserver.Z1 and Apotome (40x objective) 2h post HBV exposure to indicate YAP (green) and NF-κB (red) localization in PMHs pretreated with C29 and XMU-MP-1. Raw images were subsequently proceeded with Image J software, n=3 independent experiments **(E)**. ****p < 0.0001.

Our previous work showed that HBV infection induces the activation of TLR2 in PHHs (8). To verify whether TLR2 is required for HBV exposure-induced NF-κB and Hippo signaling in PMH, a TLR2 inhibitor (C29) was applied to PMHs 2h before HBV exposure for 6h. HBV-induced TLR signaling was obstructed in the C29-treated cells, as indicated by the decreased gene expression of *Il6* and *Tnf* compared to that in the DMSO control (**Figure 3A**). Furthermore, TLR2 blockade decreased the HBV-mediated phosphorylation of MST1/2, YAP and NF-κB (**Figure 3B**). These results imply that TLR2 is essential for the recognition of HBV particles and the activation of Hippo signaling.

MST1/2 inhibitor (XMU-MP-1) was additionally used to pretreat PMHs, prior to HBV exposure. Here, decreased phosphorylation of MST1/2, YAP and NF-κB was observed, in response to HBV treatment. Furthermore, diminished levels of phosphorylated MST1/2 impeded the intensity of HBV-induced innate immune responses, which was demonstrated by decreased *Il6* and *Tnf* expression (**Figures 3C, D**). ICC staining visualized that both, C29 and XMU-MP-1 pretreatment blocked the HBV-induced NF-κB nuclear translocation, while sustained nucleus translocation of YAP was shown (**Figure 3E**). Herein, the Hippo pathway is suggested to negatively regulate the induction of inflammatory cytokines.

### The TLR-Hippo Axis Is Functional in Mouse Model *In Vivo*

**Figure 4:**
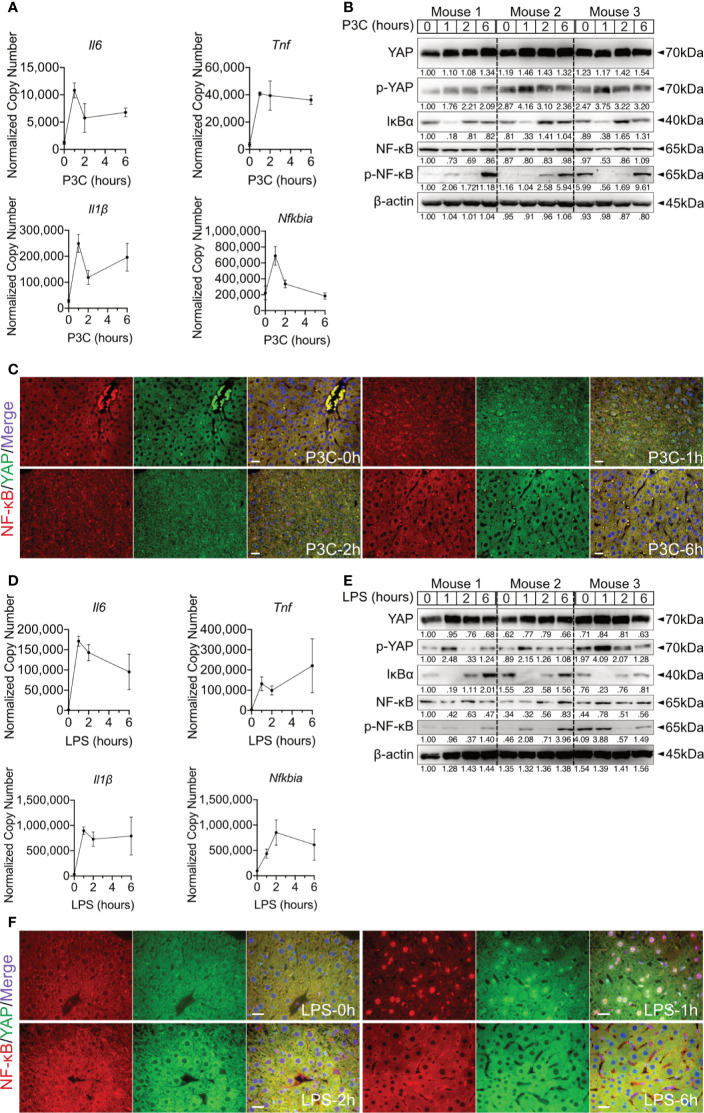
Hepatic TLR-Hippo axis plays an important role in innate immunity *in vivo*. Two-month-old male C57BL/6 mice (group size n=3) received intra venous Pam3CSK4 (P3C, 1mg/kg bodyweight) or intra peritoneal LPS (2.5mg/kg bodyweight). After 0-6h liver samples were prepared and analyzed. Quantitative PCR was performed to detect hepatic *Il6*, *Tnf*, *Il1β* and *Nfkbia* expression 0h, 1h, 2h and 6h after injection of P3C **(A)** or LPS **(D)**. Accordingly, YAP, phosphorylated YAP, IκBα, NF-κB and phosphorylated NF-κB were analyzed by western blot **(B, E)** treatment. Gel images were obtained with ADVANCED Fluorescence and ECL Imager. ICC staining was performed and images were obtained with Zeiss AxioObserver.Z1 and Apotome (40x objective) to visualize the intracellular localization of YAP (green) and NF-κB (red) at 0h, 1h, 2h and 6h after P3C **(C)** or LPS **(F)** administration. Raw images were subsequently proceeded with Image J software. Group size N=3; Scale bar, 20μm; kDa, kilodalton.

Hepatic immune induction hardly occurs in humanized liver-chimeric mice, infected with HBV (8). To investigate the hepatic TLR-Hippo axis *in vivo*, HBV-induced innate immune responses were mimicked by TLR2 ligand Pam3CSK4, administered to 2-month-old mice for different lengths of time. After mouse liver collection and appropriate sample preparation, qPCR, western blotting and ICC staining were performed to validate the previous findings *in vivo*. Quantitative PCR showed that the rapid immune response in the mouse liver occurred immediately after Pam3CSK4 injection and was indicated by a sharp increase in *Il6*, *Tnf* and *Il1β* expression (**Figure 4A**). Western blot analysis of whole liver tissue lysates (**Figure 4B**) indicated that Pam3CSK4 led to a slide induction of YAP phosphorylation 1h post exposure, while phosphorylation of NF-κB was observed 6h after treatment. The loss and rebound of IκBα 1-2h after treatment could be shown, which was consistent with the *in vitro* findings. However, ICC staining clearly showed nuclear translocation of YAP and NF-κB was observed in fixed liver tissue one hour after Pam3CSK4 administration. Thereafter, both YAP and NF-κB were gradually excluded from the nuclei (**Figure 4C**). In addition, LPS was administered to 2-month-old mice for different lengths of time. The qRT-PCR data (**Figure 4D**) and the ICC staining (**Figure 4F**) were consistent with those induced by Pam3CSK4 treatment, while western blot results slightly differed (**Figure 4E**). While Yap phosphorylation and IκBα rebound occurred, signals for NF-κB phosphorylation were inconsistent. However, the early nuclear translocation of both NF-κB and YAP was clearly indicated by ICC staining 1h after Pam3CSK4 and LPS treatment, although there were remaining cytosolic protein fractions. This subcellular distribution and the possibility that not 100% of liver cells were activated after the injection of TLR ligands might explain the weak changes obtained by western blot analysis of total liver tissue. Nevertheless, the obtained *in vivo* results confirmed our hypothesis that the TLR-Hippo axis is involved in the innate immune response in the liver.

### Hippo Signaling Regulates the Rapid Immune Response by Suppressing YAP/TEAD4-Mediated Transcription

**Figure 5:**
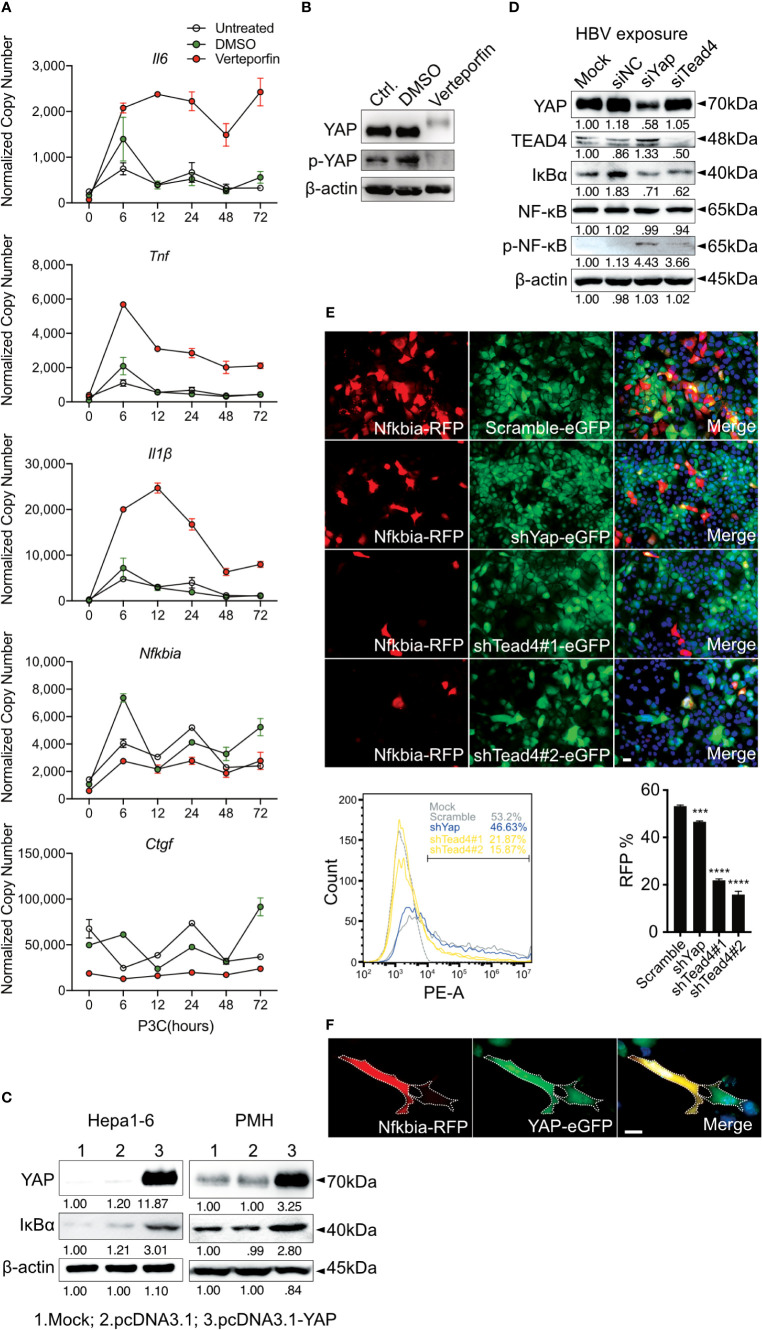
Hippo signaling regulates the inflammatory immune response by suppressing YAP/TEAD4-mediated transcription. PMHs were pretreated with Verteporfin [1μM] or DMSO for 24h and treated with Pam3CSK4 [1µg/ml] for different length of time. Gene expression of *Il6, Tnf, Il1β, Ctgf* and *Nfkbia* was determined by quantitative PCR **(A)**, (normalized to *Actb*, mean ± SEM, n=3 independent experiments). Representative YAP expression was determined by western blot in PMHs treated with Verteporfin or DMSO for 24h, n=3 independent experiments **(B)**. Western blotting revealed the expression of YAP and IκBα in Hepa1-6 cells and primary murine hepatocytes (PMHs) 48h after the overexpression of YAP, representatively. Gel images were obtained with ADVANCED Fluorescence and ECL Imager, n=3 independent experiments **(C)**. Western blot analysis detected the expression of YAP, TEAD4, IκBα, NF-κB and phosphorylated NF-κB in PMHs treated with 50nM siYap, siTead4 or siNC (non-silencing control) for 48h followed by HBV exposure for 2h, representatively. Gel images were obtained with ADVANCED Fluorescence and ECL Imager. N=3 independent experiments **(D)**. Hepa1-6 cells stably transfected with GFP-inducing shRNA plasmids (Scramble, Yap, Tead4) were co-transfected with RFP reporter plasmid to assess *Nfkbia* promoter activity. Representative ICC images and flow cytometry were applied to determine *Nfkbia* promoter-driven RFP expression with Olympus BX51 (20x objective) and CytoFLEX S flow cytometer. N=3 independent experiments **(E)**. Representative ICC images of Hepa1-6 cells showing *Nfkbia* promoter-driven RFP intensity in cells overexpressing GFP-associated YAP **(F)**. ***p-value < 0.001; ****p-value < 0.0001; DMSO, solvent control; scale bar, 20μm; kDa, kilodalton.

To confirm the regulation of inflammatory TLR signaling by the Hippo pathway, Verteporfin (1µM), a YAP/TEAD inhibitor (32), was applied to PMHs 24h before Pam3CSK4 (1µg/ml] stimulation for different lengths of time (**Figure 5A**). The inhibition of YAP/TEAD complex formation by Verteporfin treatment led to the degradation of YAP protein (**Figure 5B**) and subsequently prolonged the upregulation of cytokine expression (*Il6, Tnf* and *Il1β*) and the decrease of the *Nfkbia* and *Ctgf* gene expression. Concluding that Hippo signaling is a regulator of strength and duration of innate, inflammatory responses.

YAP/TEAD4 transcription factor complex, the effector of the Hippo pathway, plays a vital role in regulating gene transcription by recognizing TEAD4-binding sites in the promoter region of target genes. IκBα is the inhibitor of NF-κB signaling in the cytoplasm, and its expression level reveals the extent of innate immunity induced by NF-κB signaling. Because the overexpression of YAP led to increased IκBα expression in Hepa1-6 cells and PMHs (**Figure 5C**), we hypothesized that Hippo signaling reinforces the rapid innate immune response through suppressing the YAP/TEAD4-mediated expression of IκBα. To investigate this hypothesis, siRNAs against YAP and TEAD4 were utilized in PMHs. HBV exposure for 2h plainly augmented the level of phosphorylated NF-κB with a decrease in IκBα expression in PMHs with suppressed YAP or TEAD4 expression (**Figure 5D**). To confirm this transcriptional regulation, the *Nfkbia* promoter region was cloned into an RFP reporter vector and transfected into Hepa1-6 clones, stably transfected with GFP-fused shRNAs (Scramble, shYap and shTead4). Knockdown efficiencies of chosen clones are given in **Supplementary Figure 2**. The RFP expression level clearly decreased in YAP- and TEAD4-suppressed cells. These results were quantified by counting RFP-positive cells (**Figure 5E**) by flow cytometry and suggest that the YAP/TEAD4 transcription factor might compromise NF-κB signaling through promoting *Nfkbia* expression. To further confirm this mechanism of regulation, we selected a stable YAP-overexpressing Hepa1-6 clone and illustrated the overexpression of YAP by a GFP signal. After transfecting these cells with the *Nfkbia*-driven RFP reporter plasmid, we observed a more substantial RFP signal in cells exhibiting high levels of YAP than in cells expressing low levels of YAP (**Figure 5F**). Taken together, these results demonstrate that the Hippo signaling pathway can regulate the rapid innate immune response by inducing YAP/TEAD4 transcriptional activity to promote *Nfkbia* expression.

### *Nfkbia* Is a Direct Target Gene of the YAP/TEAD4 Transcription Factor Complex

**Figure 6:**
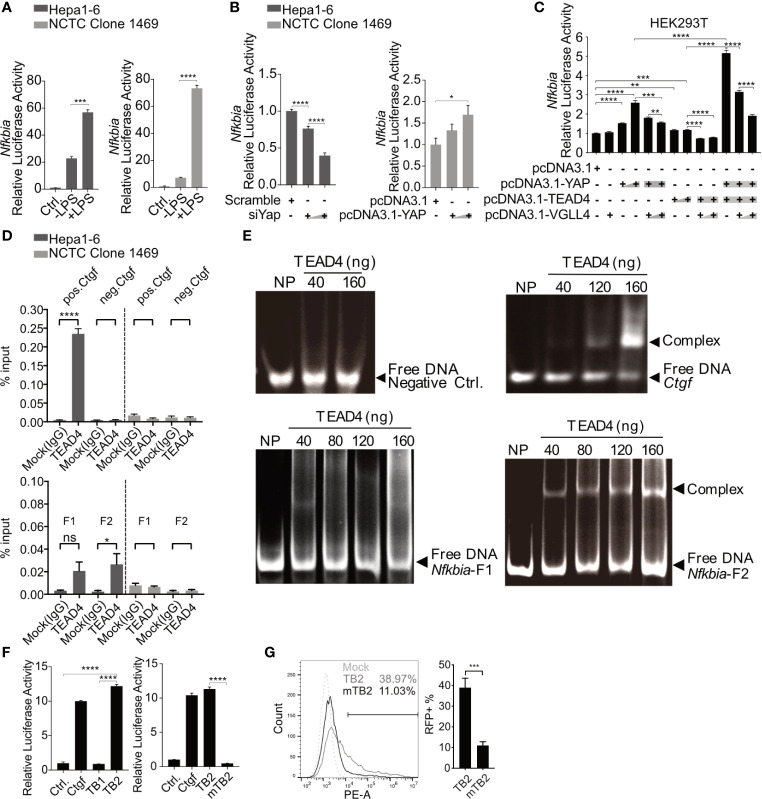
*Nfkbia* is a direct target gene of the YAP/TEAD4 complex. Reporter plasmid pGL3-Nfkbia was transfected into Hepa1-6 or NCTC clone 1469 for 12h, followed by LPS [10μg/ml] treatment for 24h and luciferase activity determination. (mean ± SEM, n=3 independent experiments) **(A)**. Dual-luciferase reporter (DLR) assay was performed to detect luciferase activity 48h after the knockdown of Yap (siYap) in Hepa1-6 cells or overexpression of YAP (pcDNA3.1-YAP) in NCTC clone 1469 cells. (mean ± SEM, n=3 independent experiments) **(B)**. DLR assay was performed after co-transfection of YAP, TEAD4 or VGLL4 overexpression plasmids and pGL3-Nfkbia in HEK293T reporter cells. (mean ± SEM, n=3 independent experiments) **(C)**. Chromatin immunoprecipitation assay was performed determining interaction between TEAD4 and the *Nfkbia* promoter **(D)** using agarose-TEAD4 antibody and sonicated chromatin (200bp-1000bp). (mean ± SEM, n=3 independent experiments). EMSA was performed to show the direct interaction of the YAP/TEAD4 complex and Nfkbia promoter by incubating TEAD4 with PCR-generated DNA fragments and subsequent gel electrophoresis, representatively. Gel images were obtained with E-box VX2 Gel Documentation Imaging system. N=3 independent experiments **(E)**. DLR assay was performed after cloning TB1, TB2 and mutated TB2 (mTB2) binding site into the reporter plasmid. (mean ± SEM, n=3 independent experiments) **(F)**. Flow cytometry was performed to detect RFP-positive cells following the transfection of TB2- or mTB2-specific RFP reporter plasmids into YAP-overexpressing Hepa1-6 cells, representatively. (mean ± SEM, n=3 independent experiments) **(G)**. *p-value < 0.05; **p-value < 0.01; ***p-value < 0.001; ****p-value < 0.0001; ns, not significant.

Through an open-source transcription factor-binding site prediction platform (http://jaspar2016.genereg.net), several TEAD4-binding sites were identified in the *Nfkbia* promoter region (-977~+34; **Supplementary Figure 3**). The promoter region was cloned into the pGL3-basic luciferase reporter vector, and DLR (dual luciferase reporter) assays were performed after TLR activation in the Hepa1-6 and NCTC clone 1469 cell lines. Lipopolysaccharide (LPS) treatment induced the Nfkbia promoter activity in both cell lines, confirming the functionality of the reporter assay (**Figure 6A**). To further investigate how the loss or gain of YAP affects *Nfkbia*-driven luciferase activity, we first knocked down YAP with siRNA in Hepa1-6 cells, and the luciferase activity was plainly decreased with an increasing dose of siRNA (**Figure 6B**). The basal YAP level was very high in the Hepa1-6 cell line; in contrast, YAP was undetectable in the NCTC clone 1469 cell line (**Supplementary Figure 4**); therefore, we decided to overexpress YAP in NCTC clone cells. The luciferase activity was upregulated with increasing YAP expression (**Figure 6B**). To carry out transcriptional activation, YAP should form a transcription factor complex with TEAD4. VGLL4 is a competitive inhibitor of YAP that binds to TEAD *via* the Tondu domain (33). In this context, YAP, TEAD4 and VGLL4 were overexpressed together in the HEK293T model cell line. In accordance with the results in the NCTC clone 1469 cell line, overexpression of YAP in the HEK293T cell line increased luciferase activity compared to the control group, but not when TEAD4 was overexpressed. Nevertheless, combined overexpression of YAP and TEAD4 strikingly increased luciferase activity (**Figure 6C**). In contrast, the luciferase activity was significantly decreased when either YAP or TEAD4 was overexpressed in combination with VGLL4. Luciferase activity was particularly reduced in a dose-dependent manner when YAP and TEAD4 were co-overexpressed with VGLL4 (**Figure 6C**). These results suggested that the YAP/TEAD4 transcription factor complex can promote *Nfkbia* expression by regulating its promoter activity.

Chromatin immunoprecipitation (ChIP) was performed to determine whether the YAP/TEAD4 transcription factor complex interacts directly with the promoter region of *Nfkbia* and which part of the promoter is bound. The entire promoter region was divided into two different clusters, fragment 1 and fragment 2, according to the distribution of TEAD4-binding sites (**Supplementary Figure 3**). Two pairs of ChIP-qPCR primers (**Supplementary Table 1**) and control primers for *Ctgf*, which is a well-known YAP/TEAD4 target gene, were designed. The ChIP results, using the positive control *Ctgf* primers and negative control primers, showed that TEAD4 can specifically bind to the *Ctgf* promoter in Hepa1-6 cells expressing high levels of YAP but not in NCTC 1469 cells expressing low levels of YAP (**Figure 6D**, upper panel). The results of ChIP with the negative control primers and a mock (IgG) experiment confirmed the quality of the ChIP assay results. The ChIP results, using specific primers for the *Nfkbia* promoter, indicated that fragment 2 (F2) seemed to be the main part of the promoter sequence, responsible for complex regulation (**Figure 6D**, lower panel).

To confirm the interaction between the transcription factor complex and fragment 2, the TEAD4 protein was overexpressed and purified. An electrophoretic mobility shift assay (EMSA) indicated that no TEAD4-DNA complex was formed with the negative control DNA fragment, which does not contain a TEAD4-binding site. *Nfkbia* fragment 2 and the *Ctgf* positive control fragment formed a complex with the TEAD4 protein in a dose-dependent manner (**Figure 6E**). The EMSA results confirm the direct interaction between the YAP/TEAD4 complex and the *Nfkbia* promoter. *Nfkbia* fragment 2 includes two TEAD4-binding sites, TB1 and TB2 (**Supplementary Figure 3**). Each TEAD4-binding motif and a 50 bp flanking sequence on each side were cloned into the pGL3-basic luciferase reporter vector, as positive control *Ctgf* promoter was cloned. A DLR assay demonstrated that TB2 is the dominant TEAD4-binding site in the *Nfkbia* promoter. Furthermore, the luciferase activity of mutated TB2 (mTB2; **Supplementary Figure 5**) was significantly decreased (**Figure 6F**). To further confirm this, the sequences of TB2 and mTB2 were cloned into the RFP reporter vector. After YAP-GFP-overexpression in Hepa1-6, cells were transfected with these vectors, RFP expression was markedly decreased in the mTB2-expressing group compared to that in the TB2-expressing group (**Supplementary Figure 6**). RFP-positive cells were counted by flow cytometry, as shown in **Figure 6G**. The percentage of RFP-positive cells was 11.03 ± 4.37% in the mTB2 group and 38.97 ± 11.24% in the TB2 group. Taken together, these results illustrated that the YAP/TEAD4 transcription factor complex can directly bind to the TB2 TEAD4-binding site located proximal to the transcription start site to promote *Nfkbia* expression.

### IRAK4 Activates MST1/2 by Phosphorylating and Inducing the Degradation of PP2A

**Figure 7:**
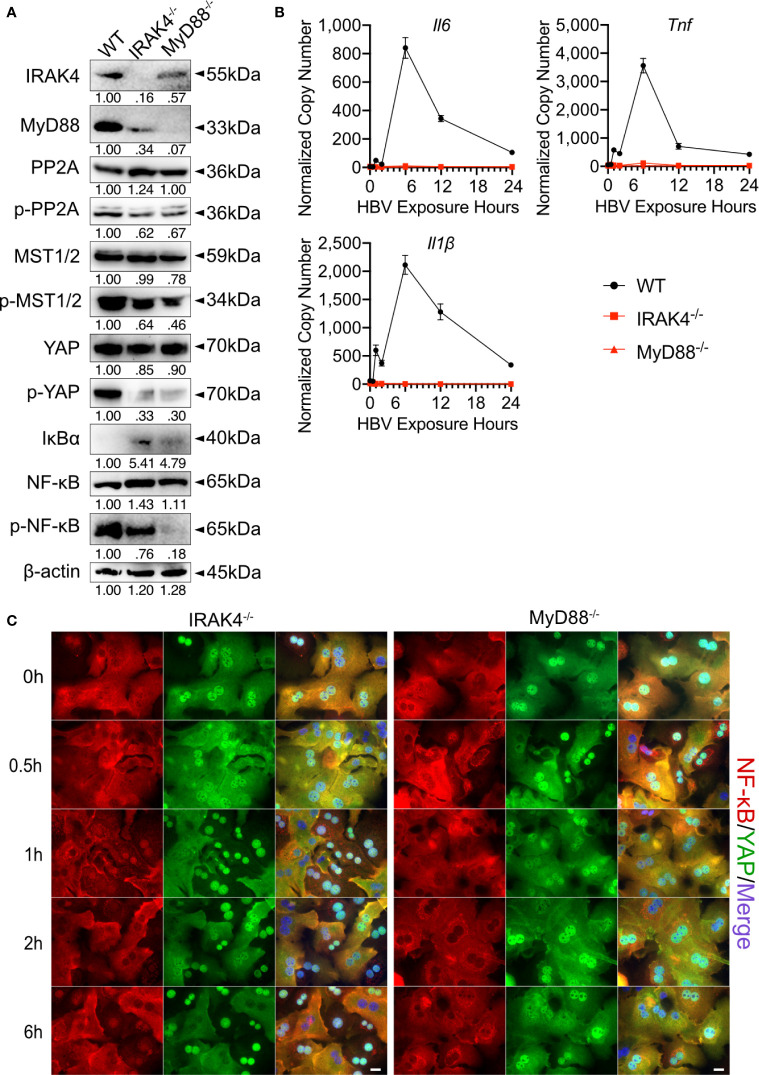
MyD88 and IRAK4 continuously activate MST1/2 by phosphorylating and inducing the degradation of PP2A. Western blotting was performed to detect the abundance of IRAK4, MyD88, PP2A, phosphorylated PP2A, MST1/2, phosphorylated MST1/2, YAP, phosphorylated YAP, IκBα, NF-κB and phosphorylated NF-κB in PMHs derived from wild-type (WT), *Irak4^-/-^* and *Myd88^-/-^* male mice, representatively. Gel images were obtained with ADVANCED Fluorescence and ECL Imager, n=3 **(A)**. Quantitative PCR was performed to detect *Il6*, *Tnf* and *Il1β* expression at different time points after the exposure of PMHs isolated from WT, *Irak4^-/-^* and *Myd88^-/-^* mice to HBV at an MOI of 1,000 **(B)**, (normalized to *Actb*, mean ± SEM, n=3). Representative ICC staining was performed and obtained with Zeiss AxioObserver.Z1 and Apotome (40x objective) to visualize the intracellular localization of YAP (green) and NF-κB (red) in PMHs derived from *Irak4^-/-^* and *Myd88^-/-^* mice after HBV exposure. Raw images were subsequently proceeded with Image J software, N=3 independent experiments **(C)**. Scale bar, 20µm; kDa, kilodalton.

In the Hippo pathway PP2A functions as a key regulator upstream of MST1/2 (20). Here, after we determined that TLR2 activates Hippo signaling after HBV exposure, the specific mechanism by which TLR2 mediates MST1/2 phosphorylation was addressed. We hypothesized that IRAK4 can increase the phosphorylation of MST1/2 by initiating the degrading PP2A (**Figure 1A**). PMHs were isolated from wild-type, *Irak4^-/-^* and *Myd88^-/-^* mice before being exposed to HBV particles for different lengths of time. Since untreated and non-particle controls didn’t induce any changes in PMHs (**Figure 2**), these two controls were omitted in followed experiments. Western blot analysis of untreated PMHs indicated, that total PP2A level was slightly elevated, while the level of phosphorylated PP2A was decreased in PMHs of *Irak4^-/-^* and *Myd88^-/-^* mice. This suggests a close interaction of IRAK4 and PP2A. Moreover, phosphorylated MST1/2, phosphorylated YAP and phosphorylated NF-κB were strikingly decreased, while IκBα was increased in PMHs isolated from *Irak4^-/-^* and *Myd88^-/-^* mouse strains (**Figure 7A**). Quantitative PCR was performed to determine *Il6, Tnf* and *Il1β* gene expression levels after HBV exposure. A lack in gene induction confirmed that NF-κB signaling was not activated in *Irak4^-/-^* and *Myd88^-/-^* -derived PMHs after HBV exposure (**Figure 7B**). ICC staining further indicated that the functional TLR-MyD88-IRAK4 axis is critical and required for Hippo signaling activation, as illustrated by the continuous nuclear transition of YAP and nuclear exclusion of NF-κB (**Figure 7C**), in the absence or presence of HBV. Taken together, these results indicate that the TLR-MyD88-IRAK4 axis might be an effective control mechanism of the Hippo pathway, mediated by the phosphorylation and therefore degradation of PP2A.

### TLR-MyD88-IRAK4-Hippo Axis Partially Explains the Hepatic Expression Profile in Patients Chronically Infected with HBV

**Figure 8:**
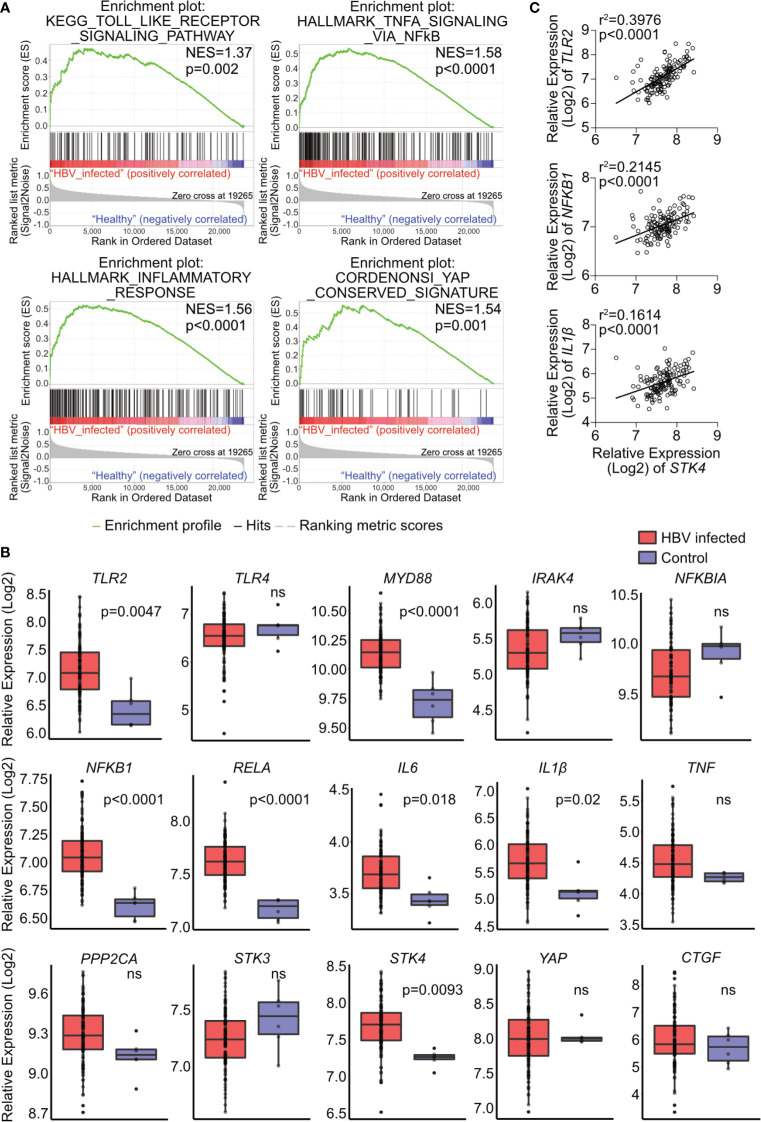
Hepatic gene expression signatures in chronic HBV-infected patients support the relevance of TLR2-MyD88-IRAK4-Hippo axis in HBV infection. GSE83148 data set (122 HBV-infected, 6 controls) was reanalyzed to perform gene set enrichment analysis **(A)**. Significant gene enrichment could be shown for TLR signaling (KEGG), NF-κB signaling and inflammatory response (Hallmark) and YAP signature (CORDENOSI). In addition, relative expression of genes related to TLR signaling (*TLR2, TLR4, MYD88, IRAK4, NFKBIA NFKB1, RELA, IL6, IL1β and TNF)* and Hippo signaling (*PPP2CA, STK3, STK4, YAP* and *CTGF*) was compared between these two groups **(B)**. Correlation of *STK4* expression (spearman) with *TLR2*, *NFKB1* and *IL1β* gene expression was performed **(C)**. ns, not significant.

To investigate the relevance of the TLR2-MyD88-IRAK4-Hippo axis in chronic hepatitis B virus infection (CHB), microarray data GSE83148 (30), including liver biopsy of 122 CHB patients and 6 uninfected controls, were reanalyzed. GSEA using hallmark, KEGG and oncogenic gene sets of the Molecular Signatures Database (v7.0) indicated significant alterations in toll-like receptor signaling, NF-κB signaling, inflammatory response and YAP signaling (**Figure 8A**). The gene expression of factors associated with the TLR2-MyD88-IRAK4-Hippo axis was compared between CHB and control samples (**Figure 8B**). In accordance with previous findings (34), hepatic *TLR2* but not the *TLR4* was upregulated in CHB patients. Intracellular effector of TLR2 signaling, *MYD88*, was upregulated in CHB patients as well, although *IRAK4* was not. The relative expression of *NFKB1*, *RELA*, *IL6* and *IL1β* was increased in the CHB group. The expression level of *STK4* was upregulated while expression of *PPP2CA*, *STK3* and *YAP* was not significantly affected. Expression of YAP/TEAD response genes *CTGF* and *NFKBIA* did not differ, when comparing CHB and control patients (**Figure 8B**). These findings led to suggest that hepatic TLR2 activation and NF-κB signaling occurred in CHB patients. Analysis of another GEO data set GSE65359 (31) allowed comparison between the different phases of chronic HBV infection. These data indicated that *TLR2*, *MyD88*, *IL1β* and *STK4* gene expression signals were significantly elevated in the immune clearance phase, compared to immune tolerant phase and inactive carrier state (**Supplementary Figure 7**). Although GSEA of GSE83148 (30) showed an enrichment of Hippo/YAP-associated genes in CHB patients (**Figure 8A**), Hippo pathway activity indicated by *CTGF* and *NFKBIA* gene expression could not be observed. This might be explained by the significantly elevated expression of *STK4* (MST1), which cytoplasmic activity negotiates YAP/TEAD transcriptional activity (**Figure 8B**). Interestingly, the expression level of *STK4* significantly correlated with those of *TLR2*, *NFKB1* and *IL1β* (**Figure 8C**). On the one hand, this observation indicates that TLR2 activation seem to occur in the immune active phase of HBV infection. On the other hand, additional regulatory mechanisms exist that control the TLR2-MyD88-IRAK4-Hippo axis *in vivo*, preventing inopportune Hippo pathway activation.

## Discussion

Chronic HBV infection is a high-risk factor for hepatocarcinogenesis. Currently, antiviral treatment is a crucial way to suppress HBV replication and with this the HBV-associated HCC prevalence. However, treatment regimens mostly do not achieve virus elimination as the desired therapeutic effect. Whether HBV activates innate immunity or represses innate host defense continues to be debated. Investigations on the mechanism of HBV evasion from innate immune monitoring are urgently needed, as novel HBV cure strategies are currently explored that include the activation of innate immune pathways (23). We here showed that (i) acute HBV infection concordantly induced TLR2 and Hippo signaling activity in primary hepatocytes, (ii) Hippo pathway activation counter regulated innate immune responses by controlling *NFKBIA* expression *in vitro* and *in vivo* and (iii) hepatic gene expression signatures related to TLR2/MyD88/NF-κB signaling occurred in CHB patients. Thorough comprehension of this interaction and possibly therapeutic intervention could improve the responsiveness and safety of immune-based antiviral treatments in patients with chronic HBV infection.

Many studies in HBV-infected patients and primate models have reported that HBV infection is unable to induce an innate immune response (4, 35–38). However, these studies either focused on interferon responses or generally assess HBV exposure for longer than 6h, after which it is difficult to detect such a rapid innate immune response. Yoneda et al. showed artefacts of inflammatory immune responses in PHHs treated with HBV for 40h (6). Here, we chose 0.5h, 1h and 2h as our main time points. Of note, we observed a rapid innate immune response after 30 min of HBV exposure that peaked between 1-2h. Nevertheless, after 6h of exposure to HBV, we observed the normalization of these inflammatory factors to basal levels, which suggests an active suppression of innate immunity. We here characterized the essential role of Hippo signaling in the innate immune response in HBV infection. Activation of MST1/2 was important for the rapid innate immune response. In addition, YAP, the effector of Hippo signaling, was crucial for balancing the over-activation of innate immunity to reduce tissue injury. The Hippo signaling pathway was originally defined as an intracellular kinase cascade coupling organ size control and tumorigenesis. Therefore, there has been long-term interest in how Hippo signaling activity is controlled in homeostasis in this field. In *Drosophila*, Hpo and Wts are key components indispensable for toll receptor-induced innate immunity *via* PII (an IRAK homolog) and MyD88 (20). However, innate immunity in mammals is much more complicated. In this study, we utilized a TLR2 inhibitor and an MST1/2 inhibitor to pretreat PMHs prior to HBV exposure. When either TLR or Hippo signaling was inhibited, downstream NF-κB signaling was substantially suppressed. Liu and Zheng et al. reported that MyD88/IRAK4, a downstream effector of TLRs, suppresses PP2A levels to activate Hippo signaling in the innate immune response (20, 39). Here, we isolated PMHs from *Myd88^-/-^* or *Irak4^-/-^* mouse livers and exposed them to HBV. Interestingly, more YAP translocated into the nucleus even without HBV exposure; moreover, no NF-κB translocated into the nucleus even with HBV exposure. This finding suggests that the TLR2-IRAK4-MyD88 axis is required for HBV particle recognition and to activate the innate immune response. MST1/2 was shown to be activated by TLR2-IRAK1/4 to regulate the expression of CXCL1 and CXCL2 during *Mycobacterium tuberculosis* infection (40). Consistent with the results of previous studies, *Irak4* or *Myd88* knockout increases PP2A levels and decreases phosphorylated MST1/2 levels, which further induces YAP translocation into the nucleus and promotes IκBα expression to repress NF-κB nuclear translocation (20). Our study is the first to report that YAP/TEAD4 directly binds to the proximal binding site of the *Nfkbia* promoter region to regulate IκBα expression. This fundamentally explains how the YAP/TEAD4 complex balances the early innate immune response.

Mouse hepatocytes do not express the sodium taurocholate co-transporting polypeptide (NTCP) receptor, the cellular factor for HBV entry (41). Here, PMHs recognized HBV particles as pathogens *via* TLR2, without the process of infection. To simulate HBV infection in mice, we treated mice with the TLR2 agonist Pam3CSK4 by i.v. injection. This method does not sufficiently represent real HBV infection but explained why the TLR2-MyD88-IRAK4-Hippo axis is important for rapid innate immunity in the liver. Future studies might utilize a humanized mouse model as an *in vivo* model for HBV infection. First approaches that addressed early immune responses in HBV-infected liver-chimeric mice neither showed nor excluded hepatic immune gene induction (8). This model needs to be adopted towards immune induction rather than stable HBV infection to enable investigations on TLR and Hippo pathway activation in the future. However, studying state of the art PHH culture led to similar results, indicating that our findings obtained in mouse models are also relevant in humans. Highlighting the importance of our study, a very recent publication reported that monocytes from chronic hepatitis B patients express higher levels of inflammatory cytokines than those from healthy donors. HBV induces the production of inflammatory cytokines *in vitro via* TLR2/MyD88/NF-κB signaling in monocytes (42). Furthermore, reanalysis of large-scale gene expression data (30) of liver biopsies, led to suggest an increased hepatic TLR2/MyD88/NF-κB signaling in CHB patients. The control of Hippo signaling in long-term persistent HBV-infection likely protects from oncogenic processes. These findings may have implications for novel HBV cure strategies. Immunotherapeutics being under clinical development include MyD88-dependent TLR7 and TLR8 as well as RIG-I agonists (SB9200) (23). Whether and how these ligands affect the Hippo pathway, thereby contributing to immune control and tumorigenesis is an urgent and open question.

In summary, we demonstrate that HBV-mediated activation of TLR2, which not only induced NF-κB signaling but also activated the Hippo pathway to regulate the innate immune response by producing and controlling inflammatory factors. YAP/TEAD4 functioned as a negative regulator and is important for balancing innate immune response *via* induction of IκBα. Consistent with reports that HBV is a “stealth virus”, our study indicated a rapid innate immune response at a very early stage after HBV exposure that is subsequently suppressed by IκBα and further unknown mechanism that might relate to HBV evasion from innate immunity. This rapid innate immune response is required for host defense but is a double-edged sword as it also induces tissue injury when activation is too strong. Activation of the Hippo signaling effector YAP accompanied the TLR2-NF-κB-mediated immune response and negatively regulates its activation to reduce tissue injury. Taken together, our data indicate that the Hippo signaling pathway harbors a regulatory task in the rapid innate immune response. These findings directly link the hepatic inflammation with growth control mechanisms and might explain tumor progression in non-cirrhotic HBV-infected patients.

## Data Availability Statement

The raw data supporting the conclusions of this article will be made available by the authors, without undue reservation.

## Ethics Statement

The studies involving human participants were reviewed and approved by institutional review board (ethics committee) of the medical faculty at the University Duisburg-Essen. The patients/participants provided their written informed consent to participate in this study. The animal study was reviewed and approved by Landesamt für Natur, Umwelt und Verbraucherschutz Nordrhein-Westfalen.

## Author Contributions

The main study was conceptualized by XL and RB, in parts by SL and RZ. Methodology and experimental setup were performed by XL, ML, and HB. The original draft was written by XL and RB. Manuscript discussion, review and editing were performed by RZ, SL, ML, GG, and HW. RB supervised the project. All authors contributed to the article and approved the submitted version.

## Funding

RB received funding from the Deutsche Forschungsgemeinschaft (DFG: TRR60, BR4014/5-1 & BR4014/8-1). RB received internal funds (Programm zur internen Forschungsförderung Essen, IFORES), and GG and HW provided intramural funds.

## Conflict of Interest

The authors declare that the research was conducted in the absence of any commercial or financial relationships that could be construed as a potential conflict of interest.
