# Supplementary material for: Hippo Pathway Counter-Regulates Innate Immunity in Hepatitis B Virus Infection
Source: Front Immunol. 2021 May 25;12:684424. doi: 10.3389/fimmu.2021.684424 (PMC8185339; doi:10.3389/fimmu.2021.684424)
Supplement: Supplementary file 1 [file DataSheet_1.pdf]

Supplementary Material

Hippo pathway counter-regulates innate immunity in Hepatitis B virus infection

Xufeng Luo, Rui Zhang, Mengji Lu, Shi Liu, Hideo A. Baba, Guido Gerken, Heiner Wedemeyer, Ruth Broering

Correspondence to: ruth.broering@uni-due.de

This PDF file includes:

Supplementary Figures 1 to 8
Supplementary Tables 1 to 4

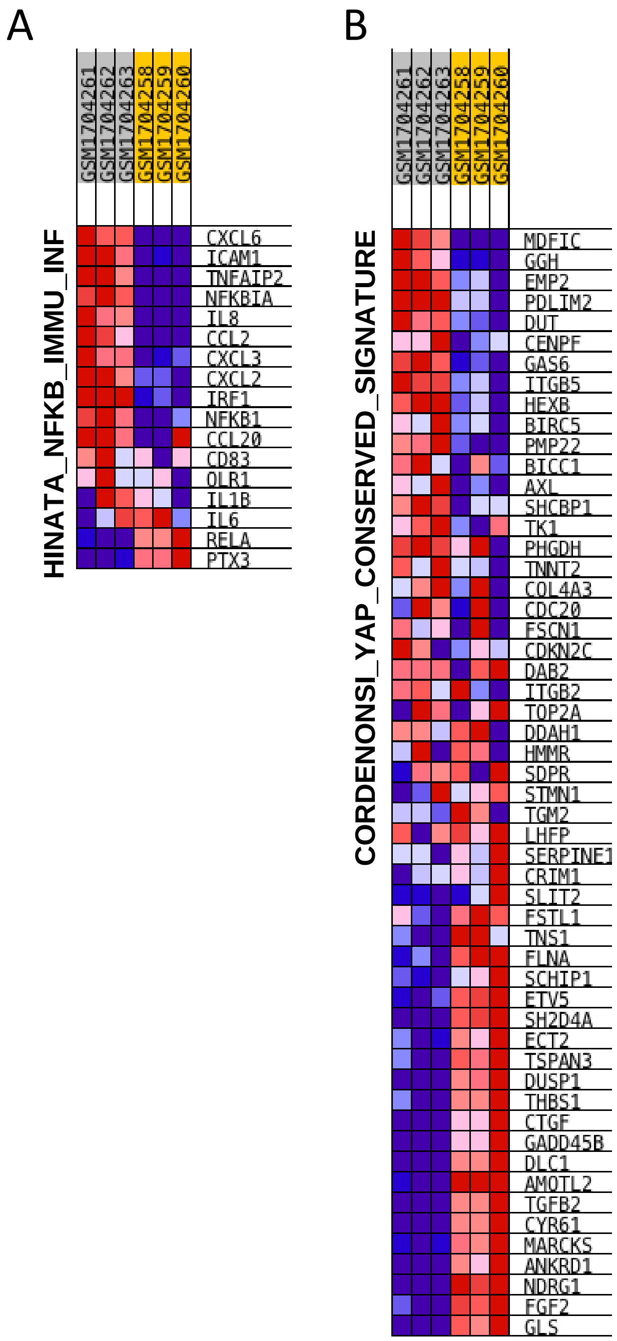

Supplementary Figure 1. HBV infection induces NF-κB-related genes and shifts YAP-conserved gene expression in primary human hepatocytes. In GSE69590 microarrays of PHHs, infected with HBV particles at an MOI of 50 (GSM1704261, GSM1704262, GSM1704263, shaded in grey) for 40h or untreated control (GSM1704258, GSM1704259, GSM1704260, shaded in yellow), were performed. Gen set enrichment analysis of GSE69590 GEO data set (matrix based on log2 values) identified the enrichment of genes related to HINATA_NFKB_IMMU_INF (A) and CORDENONSI_YAP_CONSERVED_ SIGNATURE (B). Get sets were visualized in heatmaps indicating gene IDs and signal intensities. Expression values are represented as colors, where the range of colors (red, pink, light blue, dark blue) shows the range of expression values (high, moderate, low, lowest).

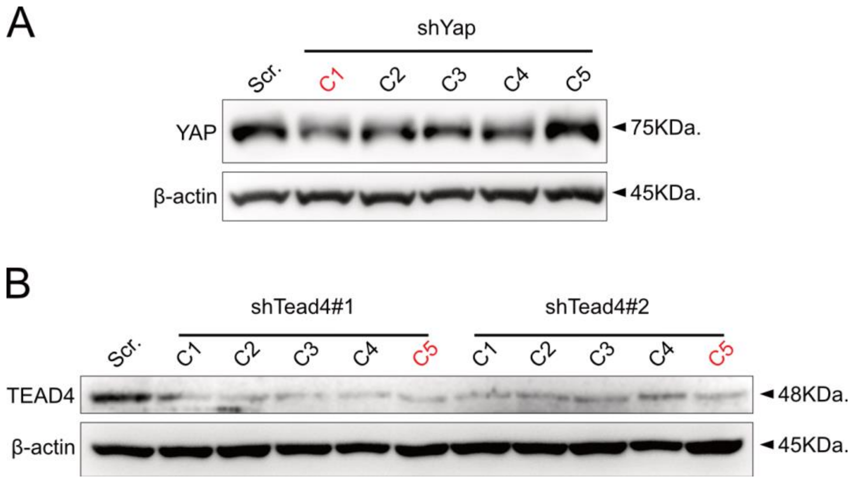

Supplementary Figure 2. **Effective knockdown subclones were selected after transfected shRNA-overexpressing vectors in Hepa1-6 cells**. The psiRNA-shYap and psiRNA-shTead4#1/#2 were transfected into Hepa1-6 cells, respectively, culture medium was supplemented with Zeocin (1000μg/ml) after 24 hours transfection. Keep selecting for one week. Selected Hepa1-6 were diluted and seeded in 96-well plates to obtain subclone. Down regulation of YAP was detected by Western blot, shYap-C1 subclone was selected for further experiments (A). Down regulation of TEAD4 was detected by Western blot, shTead4 #1C5 and shTead4 #2C5 subclones were selected for further experiments (B). Gel images were obtained with ADVANCED Fluorescence and ECL Imager. kDa, kilodalton.

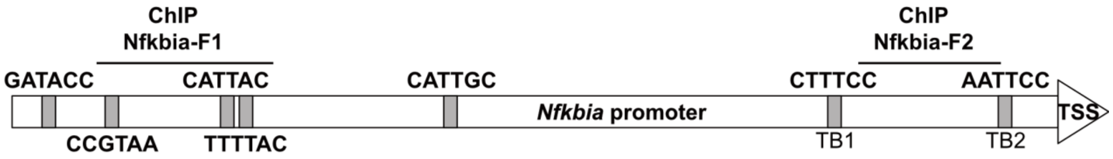

Supplementary Figure 3. Scheme for YAP/TEAD4 binding sites in the *Nfkbia* promoter region (-977~+34, NM_010907). Grey boxes represent predicted TEAD4 binding sites (TB), the upper two lines showed the ChIP-qPCR products which were produced by two pair of specific ChIP primers. Two proximal binding sites were named as TB1 and TB2. TSS, transcription start site.

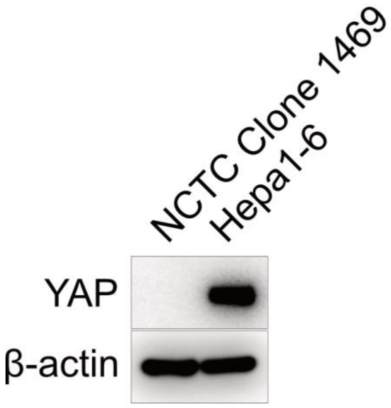

Supplementary Figure 4. **The abundance of YAP was different in NCTC Clone 1469 and Hepa1-6 cell lines.** Western blot was performed to determine YAP expression in Hepa1-6 and NCTC Clone 1469 cell lines. Gel images were obtained with ADVANCED Fluorescence and ECL Imager.

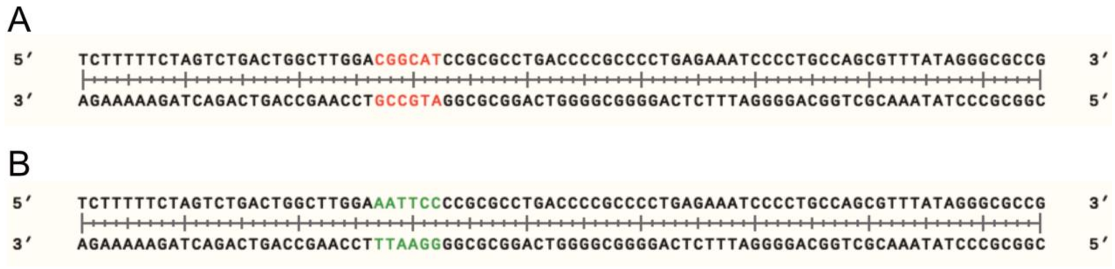

Supplementary Figure 5. **Wildtype TB2 and mutant TB2 sequence (-26~-21) information.** Red letters showed the mutant TB2 (A). Green letters showed the wildtype TB2 (B).

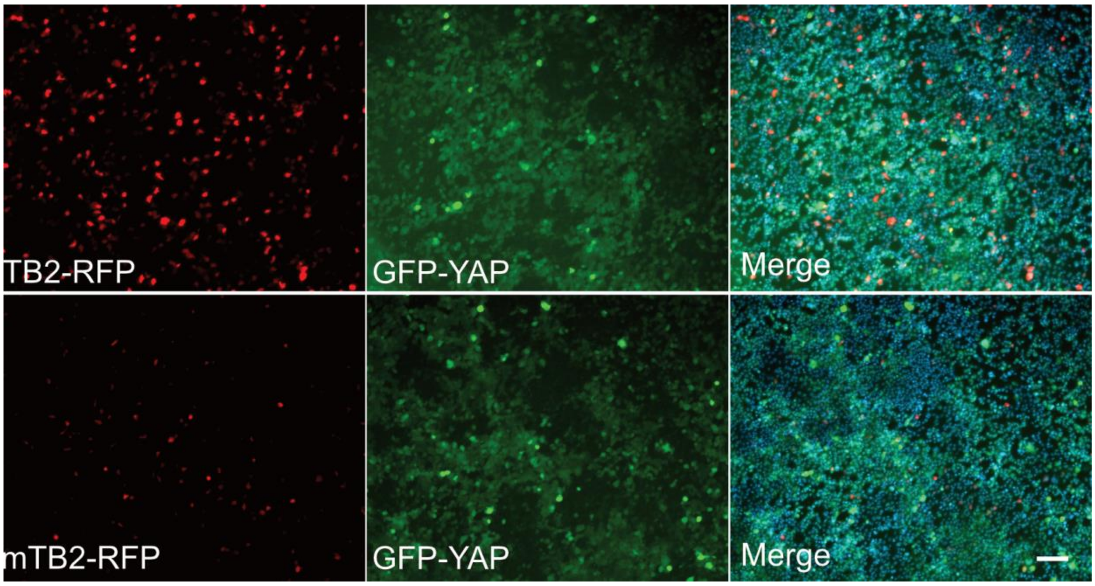

Supplementary Figure 6. Mutated TB2 binding site weakened RFP signals in YAP-GFP-overexpressed Hepa1-6 cells. TB2 and mTB2 binding sites were cloned into RFP reporter plasmid, respectively. Plasmids were transfected into YAP-GFP-overexpressed Hepa1-6 cells. After 48 hours, RFP signals were observed with Zeiss AxioObserver.Z1 and Apotome (10x objective). Scale bar is 100μm.

A

KEGG_TOLL_LIKE_RECEPTOR_SIGNALING_PATHWAY

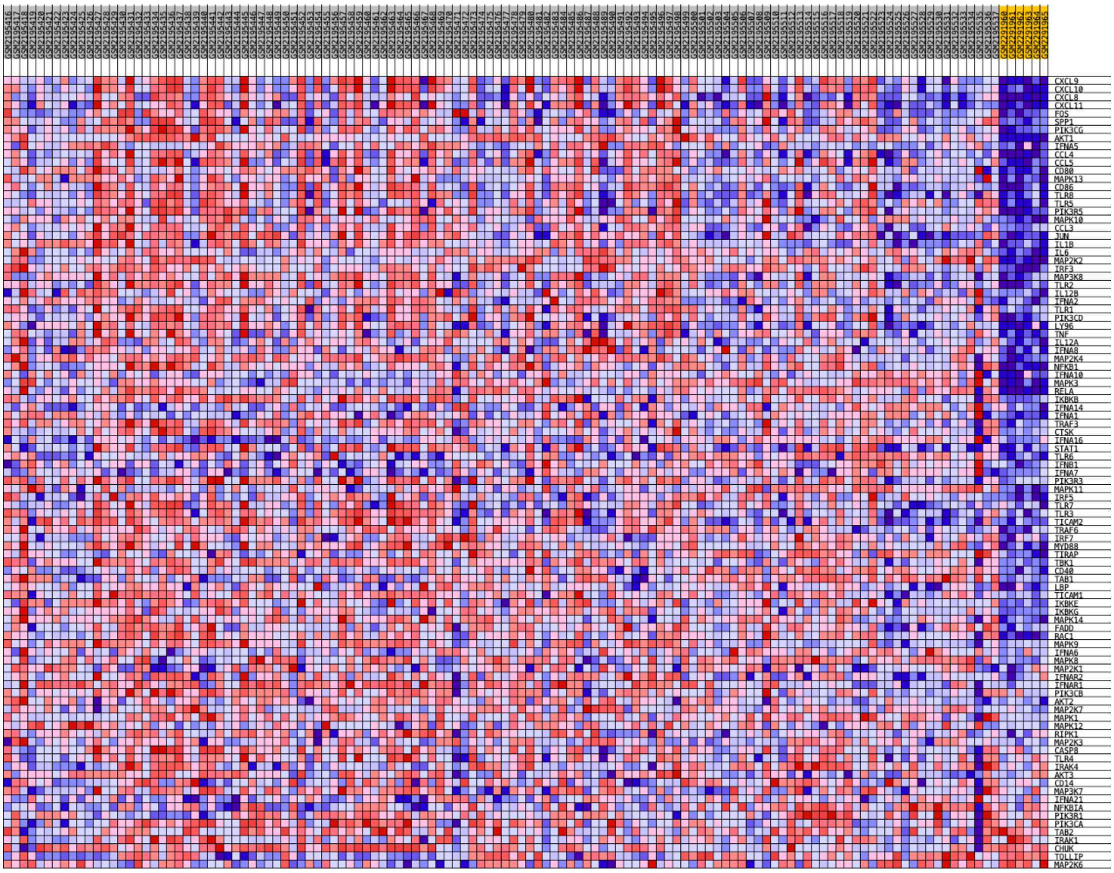

B

HALLMARK_TNFA_SIGNALING_VIA_NFKB

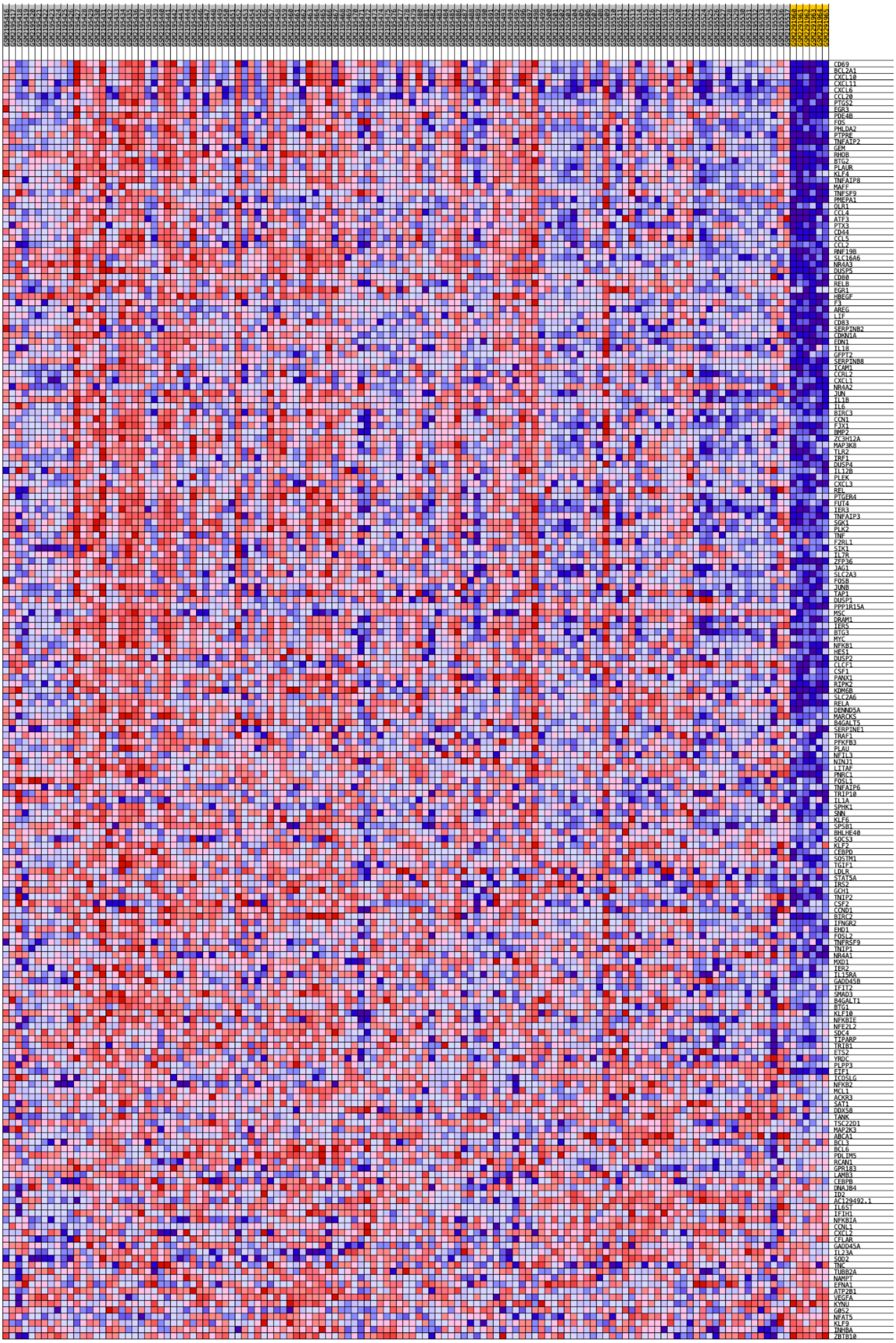

C

HALLMARK_INFLAMMATORY_RESPONSE

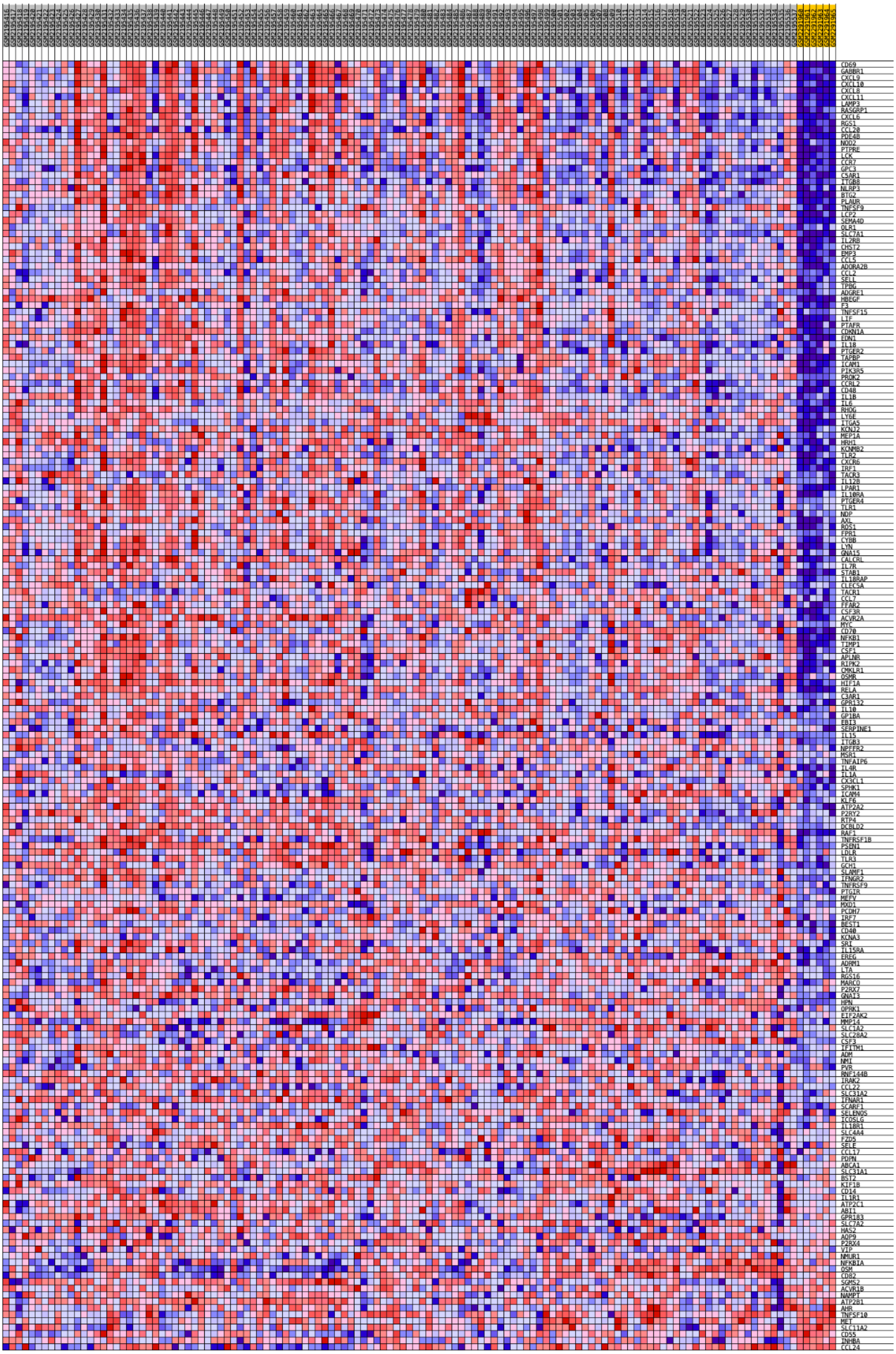

D

CORDENONSI_YAP_CONSERVED_SIGNATURE

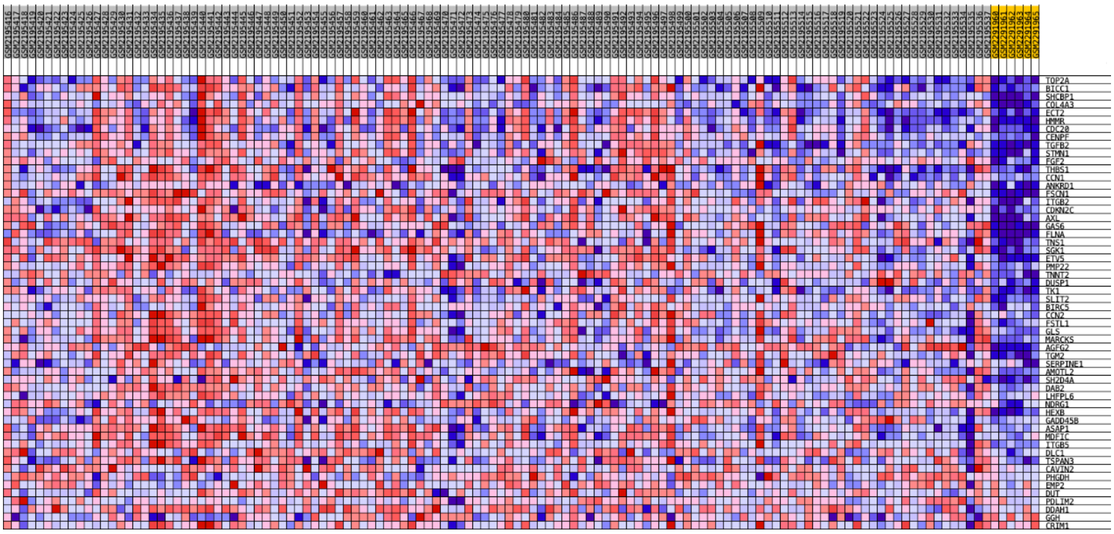

Supplementary Figure 7. HBV infection induces TLR responses, inflammatory signaling and YAP-conserved gene expression in patients with chronic hepatitis B. In GSE83148 hepatic gene expression was analyzed by microarray including liver biopsies of 122 HBV-infected patients (shaded in grey) and 6 uninfected controls (shaded in yellow). Gen set enrichment analysis of GSE83148 GEO data set was performed (matrix based on log2 values). Following gen sets

A) KEGG_TOLL_LIKE_RECEPTOR_ SIGNALING_PATHWAY
B) HALLMARK_TNFA_SIGNALING_VIA_NFKB
C) HALLMARK_INFLAMMATORY_RESPONSE
D) CORDENONSI_YAP_ CONSERVED_SIGNATURE

were visualized in heatmaps indicating gene IDs and signal intensities. Expression values are represented as colors, where the range of colors (red, pink, light blue, dark blue) shows the range of expression values (high, moderate, low, lowest).

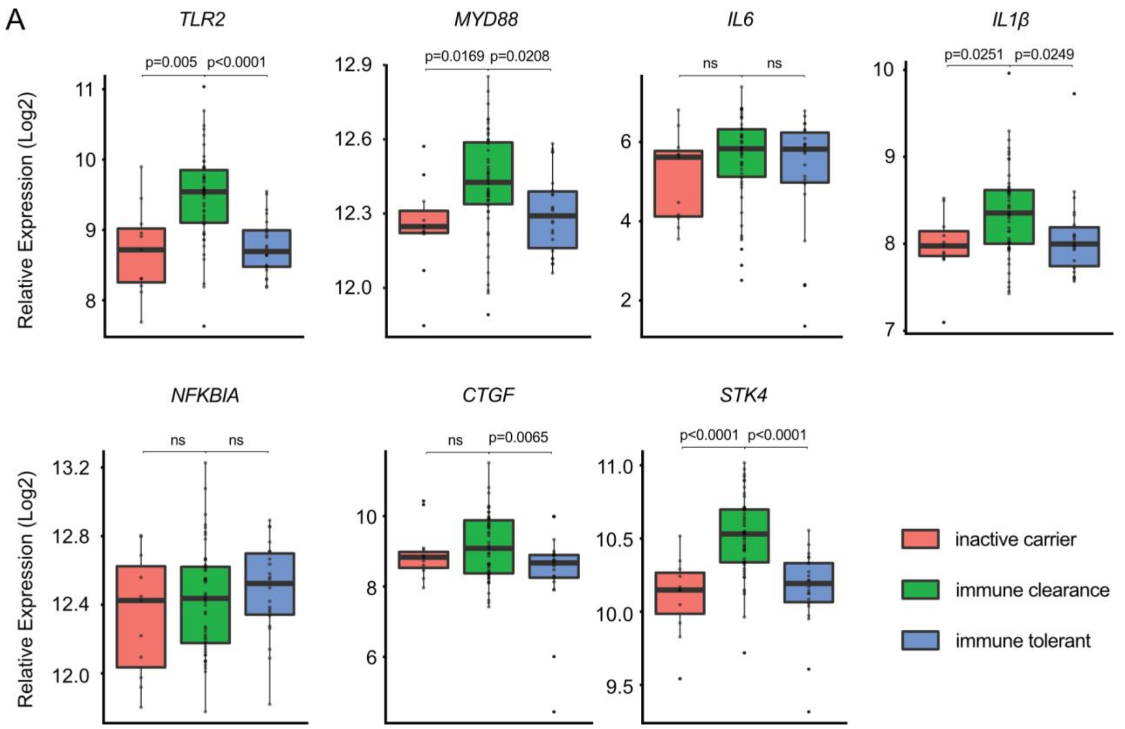

Supplementary Figure 8. *TLR2*-*MYD88*-*STK4* axis was activated in immune clearance phase of chronic HBV infection. GSE65359 data set was reanalyzed to show relative expression of genes. 83 chronic hepatitis B patients (22 immune tolerant, 50 immune clearance, and 11 inactive carrier state) were included in the data set. *TLR2*, *MYD88*, *IL6*, *IL1β*, *NFKBIA*, *CTGF* and *STK4* mRNA level were reanalyzed in each group (A).

Supplementary Table 1. siRNA and shRNA sequences

siRNA/shRNA	Target sequence (5'-3')	mRNA accession no.
Non-silencing control (NC)	CAACAAGATGAAGAGCACCAA-dT-dT	-
Yap	CTGGTCAAAGATACTTCTTAA-dT-dT	NM_009534
Tead4#1	GCTGAAACACTTACCCGAGAA-dT-dT	NM_011567
Tead4#2	CCCTCTCTGTGAGTACATGAT-dT-dT	NM_011567

Supplementary Table 2. ChIP primer

Gene	Sequence (5'-3')
Nfkbia-fragment1-forward	TTGCTCTGCTAGGCATTCACAA
Nfkbia-fragment1-reverse	CCGTATGGGAACCACATTTTTC
Nfkbia-fragment2-forward	TCAAAAAGTTCCCTGTGCATGA
Nfkbia-fragment2-reverse	CCAAGCCAGTCAGACTAGAAAAAGA
neg-control-*Ctgf*-forward	CAGTGGAGATGCCAGGAGAAA
neg-control-*Ctgf*-reverse	CCCCGGTTACACTCCAAAAA
pos-control-*Ctgf*-forward	CTTCTTGGTGTTGTGCTGGAAAC
pos-control-*Ctgf*-reverse	GACCCCTTGACACTCCACATTC

neg, negative; pos, positive.

Supplementary Table 3. Quantitative PCR primer

Gene	Sequence (5'-3')	mRNA accession no.
mActb forward	AAATCGTGCGTGACATCAAA	NM_007393
mActb reverse	CAAGAAGGAAGGCTGGAAAA	
mNfkbia forward	TCCTGCAGGCCACCAACTA	NM_010907
mNfkbia reverse	TCAGCACCCAAAGTCACCAA	
mCtgf forward	GTGTGCACTGCCAAAGATGGTGC	NM_010217
mCtgf reverse	GCACGTCCATGCTGCACAG	
hACTB forward	TCCCTGGAGAAGAGCTACGA	NM_001101
hACTB reverse	AGCACTGTGTTGGCGTACAG	
hYAP forward	GGTTGGGAGATGGCAAAGAC	NM_001130145
hYAP reverse	GGGTCCTGCCATGTTGTTGT	
hNFKBIA forward	GAGACCTGGCCTTCCTCAACT	NM_020529
hNFKBIA reverse	TTCTGGCTGGTTGGTGATCA	
hCTGF forward	GGAGCGCCTGTTCCAAGAC	NM_001901
hCTGF reverse	CTGCAGGAGGCGTTGTCATT	

m, murine; h, human.

Expression of murine *Il6, Il1β, Tnf* and human *IL6, IL1β*, *TNF* was detected by commercially available primer sets (QuantiTec Primer Assay, Qiagen; sequences are not given by the manufacturer).

Supplementary Table 4. Antibodies for immunoblot, immunostaining and ChIP

Name	Dilution	Source
YAP	WB: 1:1000	Cell Signaling, #4912
p-YAP	WB: 1:1000	Cell Signaling, #4911
PP2A	WB: 1:1000	Cell Signaling, #2038
p-PP2A	WB: 1:200	Santa Cruz, # sc271903
NF-κB	WB: 1:1000	Cell Signaling, #8242
IκBα	WB: 1:100	Santa Cruz, # sc1643
p-NF-κB	WB: 1:1000	Cell Signaling, #3033
IRAK4	WB: 1:1000	Cell Signaling, #4363
MyD88	WB: 1:1000	Cell Signaling, #4283
MST1	WB: 1:1000	Cell Signaling, #3682
MST2	WB: 1:1000	Cell Signaling, #3952
p-MST1/2	WB: 1:1000	Cell Signaling, #49332
β-actin	WB: 1:2000	Cell Signaling, #3700
Anti-Rabit-IgG	WB: 1:2000	Cell Signaling, #7074
anti-Mouse-IgG	WB: 1:2000	Sigma, #4416
Donkey Anti-Rabbit IgG-Alexa Fluor 488	ICC: 1:2000	Abcam, #ab150073
Donkey anti-Mouse IgG Highly Cross-Adsorbed-Alexa Fluor 594	ICC: 1:500	Invitrogen, #A21207
YAP	ICC: 1:100	Cell Signaling, #14074
NF-κB	ICC: 1:800	Cell Signaling, #6956
TEAD4	ChIP: 10μg/IP<br>WB: 1:500	Abcam, # ab58310

WB, Western blot; ICC, immunocytochemistry; ChIP, chromatin immunoprecipitation.
